# Comparative antibacterial and anti-virulence effects of silver ions from electrolysis, silver nanoparticles, and silver nitrate against *Pseudomonas aeruginosa* and *Staphylococcus aureus*

**DOI:** 10.1038/s41598-025-34914-3

**Published:** 2026-02-02

**Authors:** Marwa M. Eltarahony, Mohamed E. Abughulah, Mamdouh M. Shawki, Amira Sabry, Moataz M. Fahmy

**Affiliations:** 1 Environmental Biotechnology Department, Genetic Engineering and Biotechnology Research Institute, City of Scientific Research and Technological Applications (SRTA-City), Alexandria, Egypt; 2 Medical Biophysics Department, Medical Research Institute, Alexandria University, Alexandria, Egypt; 3 Protein Research Department, Genetic Engineering and Biotechnology Research Institute (GEBRI), City of Scientific Research and Technological Applications (SRTA-City), Alexandria, Egypt

**Keywords:** Silver ions, Silver nanoparticles, Electrolysis, Antibacterial activity, *Pseudomonas aeruginosa*, *Staphylococcus aureus*, Biochemistry, Biotechnology, Microbiology, Nanoscience and technology

## Abstract

The biological activity of silver-based antimicrobial agents is strongly influenced by the source and generation method of silver ions (Ag⁺); however, a direct comparison of their efficacy from different sources has not been previously reported. In this study, we systematically compared the antibacterial and anti-virulence effects of Ag⁺ derived from three sources: direct current (DC) electrolysis, silver nanoparticles (Ag-NPs), and silver nitrate (AgNO₃), standardized to 400 ppm. Electrolyzed Ag⁺ exhibited the strongest antibacterial effect, reducing viable counts of *Pseudomonas aeruginosa* and *Staphylococcus aureus* by 91.06% and 71.10%, respectively. It also caused substantial membrane disruption, reduced motility, and increased cellular leakage, as confirmed by SEM imaging, DNA and K⁺ ion leakage, and extracellular protein release. Furthermore, electrolyzed Ag⁺ most effectively suppressed key virulence factors, including biofilm formation, pigment production (pyocyanin, staphyloxanthin), and the activities of protease, esterase, and hemolysin. Despite identical Ag⁺ concentrations, the biological activity varied significantly with ion source, underscoring the importance of the delivery mechanism. These findings highlight the superior efficacy of electrolyzed Ag⁺ and support its potential application in clinical, environmental, and industrial antimicrobial strategies.

## Introduction

Infectious diseases caused by microbial pathogens remain a leading cause of morbidity and mortality worldwide, as reported by the World Health Organization (WHO)^1^. Among the most clinically significant bacteria are *Pseudomonas aeruginosa*, a Gram-negative pathogen frequently associated with respiratory and nosocomial infections, and *Staphylococcus aureus*, a Gram-positive organism responsible for a wide range of community and hospital-acquired infections^2,3^. Both species belong to the highly virulent and drug-resistant ESKAPE group, comprising pathogens prioritized for urgent therapeutic development due to their growing resistance to conventional antibiotics^2^.

The global rise of multidrug-resistant bacteria has prompted an urgent need for alternative antimicrobial strategies. A recent meta-analysis estimated that antibiotic resistance was associated with 4.95 million deaths in 2019, underscoring the severity of the issue and the demand for effective countermeasures^4^.

Nanomaterials have attracted considerable interest in this context due to their unique physicochemical properties, including nanoscale size and high surface area, which enable antibacterial effects through mechanisms distinct from those of traditional antibiotics. Among these, silver nanoparticles (Ag-NPs) have been widely studied for their broad-spectrum antibacterial activity, biofilm inhibition, and oligodynamic effect even at low concentrations^5^. Their antimicrobial efficacy is largely attributed to their capacity to release silver ions (Ag⁺), which interact with bacterial DNA, membrane proteins, enzymes, and cofactors, thereby disrupting essential cellular functions^6^.

Although silver ions are widely used as antimicrobial agents, their biological performance may vary considerably depending on their physicochemical form and method of generation, which influence ion availability, oxidative state, and biological interaction^7^. Electrolyzed silver, in particular, has gained interest as a clean and controllable ion source, yet it remains underexplored compared to silver salts and nanoparticles^8^. Moreover, while previous studies have investigated individual silver formulations, no direct comparison has been conducted to assess the antibacterial and anti-virulence efficacy of Ag⁺ ions derived from electrolysis, Ag-NPs, and AgNO₃ under equivalent concentration conditions^9^.

This study fills this gap by systematically evaluating and comparing silver ions from three sources: electrolyzed Ag⁺, silver nanoparticles, and silver nitrate against *P. aeruginosa* and *S. aureus*, two clinically relevant and highly virulent pathogens. By standardizing the silver ion concentration (400 ppm) across all treatments, we aimed to isolate the effect of ion generation and provide a clear comparison of antimicrobial and anti-virulence properties. These findings shed new light on the molecular mechanisms of silver ion action, with the aim of guiding the rational design of silver-based biomaterials for infection prevention and clinical use.

## Materials and methods

### Preparation and growth conditions of the examined pathogens

This study employed two reference bacterial strains: *Pseudomonas aeruginosa* (ATCC 27,853), representing Gram-negative bacteria, and *Staphylococcus aureus* (ATCC 25,923), representing Gram-positive bacteria. Both strains were cultured on nutrient agar plates and incubated aerobically at 37 °C for 16–24 h.

### Characterization of commercially obtained Ag-NPs

Silver nanoparticles (Ag-NPs; 1 mg/mL) were purchased from Nawah Scientific (Cairo, Egypt). The nanoparticles were dispersed in ultrapure water prior to analysis. During handling, Ag-NPs suspensions were protected from direct light by wrapping containers in aluminum foil to minimize photo-oxidation. The following characterizations were performed:

#### Electron microscopy (TEM/SEM)

 Surface morphology and particle size were analyzed using transmission electron microscopy (TEM) and scanning electron microscopy (SEM) (ZEISS, Germany).

#### UV–Vis spectroscopy

The absorbance spectrum of the Ag-NPs was recorded using a T60 UV–Vis spectrophotometer (Pusa, Japan).

#### Zeta potential measurement

The surface charge and colloidal stability of the Ag-NPs were assessed using a Zetasizer Nano ZS (Malvern Instruments, UK).

#### X-ray diffraction (XRD)

Crystallographic structure was analyzed using an X’Pert PRO powder diffractometer (PANalytical, Netherlands) with Cu Kα radiation (λ = 1.5406 Å), scanning range of 2θ = 20°–80°, and a step size of 0.001°.

#### Fourier transform infrared spectroscopy (FTIR)

Functional groups on the Ag-NPs surfaces were identified using a Bruker FTIR spectrometer (Germany), scanned in the range of 4000–500 cm⁻^1^.

### Preparation of silver ions (Ag +) samples

#### Silver ions (Ag +) generated by electrolysis

Electrolyzed silver ions were generated via a direct current (DC) electrolysis system using silver electrodes. The electrolysis was performed using two rectangular Ag/AgCl electrodes (each 1 cm × 5.0 cm, with a total active area of 10 cm^2^) positioned 2 cm apart. The electrodes were placed in parallel inside a sterile polypropylene conical tube containing distilled water as the electrolyte. The electrodes were connected to a regulated DC power supply (Etommens eTM-305A, China) delivered 5–15 V, and the resulting current was monitored continuously, ranging from 15 at 5 V to 45 mA at 15 V. The electrolyte (50 mL distilled water) was gently stirred using a magnetic bar (200 rpm) throughout electrolysis to ensure uniform ion dispersion. All procedures were conducted under ambient laboratory light with no direct sunlight exposure.

The Ag/AgCl electrodes used were factory-coated with a thin AgCl layer that served as the chloride source, ensuring stable electrode potential even in distilled water. No additional chloride salts were added to avoid ionic contamination. The solution’s pH and conductivity were measured before and after electrolysis (initial pH = 6.8; final pH = 7.1; conductivity increased from 1.2 µS/cm to 18.5 µS/cm), confirming minimal alteration of medium neutrality and moderate ion enrichment. Reactive oxygen species (ROS) formation was not quantified in this study but will be examined in future work to better elucidate electrolysis-induced oxidative mechanisms.

#### Silver ions generated by dissolving silver compounds

Silver ions were also prepared by dissolving 0.25, 0.5, and 1 gm of silver nitrate (AgNO₃) in 50 mL of distilled water. The solutions were stirred until the silver nitrate was completely dissolved.

### Ions concentration determination

The silver ion concentrations in samples generated by electrolysis, silver nanoparticles, and silver nitrate were measured using inductively coupled plasma optical emission spectrometry (ICP-OES). Prior to analysis, all silver preparations were passed through 0.2 µm syringe filters (Whatman, UK) to remove particulate and aggregated silver species. This routine ultrafiltration step ensures that the subsequent measurement represents the soluble silver fraction, composed primarily of ionic Ag⁺. During analysis, samples were nebulized and introduced into the plasma, where a high-frequency electromagnetic field excites the atoms and ions, causing them to emit light at characteristic wavelengths. A diffraction system disperses the emitted light into its spectral components, which are detected and quantified to determine elemental concentrations^10^.

### Bacterial culture exposure

A loopful of each bacterial strain was inoculated into 50 mL of Luria–Bertani (LB) broth and incubated for 24 h at 37 °C in a rotary shaker incubator set at 150 rpm. After incubation, bacterial suspensions reached a concentration of approximately 1 × 10⁹ CFU/mL. These suspensions were then exposed to electrolyzed silver ions, silver nanoparticles, and silver ions from silver nitrate. The same silver ion concentration (400 ppm) was used for all treatments to ensure comparability. Untreated cells served as the control.

### Effects of treatment conditions on microorganisms

#### Cultivability assessment

The pour plate method was employed to determine the bacterial count (CFU/mL) for each strain. Briefly, 1 mL of serially diluted bacterial suspensions, was mixed with molten agar maintained at 40–50 °C. The mixture was gently swirled in both clockwise and counterclockwise directions to ensure uniform distribution, then poured into sterile Petri dishes. After solidification, plates were incubated at 37 °C for 24 h. Following incubation, the number of viable colonies was counted, and the percentage of growth inhibition was calculated using a previously described formula^11^.

1 $${\text{Growth inhibition }}\left( \% \right) = \left[ \begin{gathered} \left( {{\text{Number of colonies in control}} - {\text{Number of colonies after treatment}}} \right) \hfill \\ / \left( {\text{Number of colonies in control}} \right) \hfill \\ \end{gathered} \right] \times {1}00$$

#### Metabolic Activity Determination

The MTT reduction assay was used to assess bacterial metabolic activity^12^. A stock solution of MTT (5 mg/mL) was prepared in sterile distilled water and filtered through a 0.22 µm membrane. MTT is reduced by metabolically active cells to insoluble purple formazan crystals. For treatment, 20 µL of each bacterial suspension (10⁸ CFU/mL) was added to 40 mL of nutrient broth. After incubation, cultures were centrifuged at 4000 × g for 3 min to collect the formazan pellet, which was resuspended in DMSO. Absorbance (A) at 570 nm was measured, and inhibition was calculated as:

2 $${\text{Inhibition }}\left( \% \right) = \left[ {{1} - \, \left( {{\mathrm{A}}_{{{\mathrm{Treated}}}} /{\mathrm{A}}_{{{\mathrm{Control}}}} } \right)} \right] \times {1}00$$

#### Sliding motility assay

Sliding motility was assessed on low-agar (0.3%) LB medium as described by Rashid and Kornberg^13^. Briefly, 5 µL of overnight bacterial cultures (~ 10⁸ CFU/mL) of *P. aeruginosa* and *S. aureus* were spot-inoculated at the center of the plates and incubated at 30 °C for 24 h. The diameters of the spreading zones were then measured in millimeters. Variations in sliding motility were interpreted relative to untreated control groups.

#### DNA leakage

Membrane integrity was evaluated by measuring extracellular DNA release, following the method of Wu and Xi^14^. Bacterial cultures (~ 10⁸ CFU/mL) were incubated at 37 °C. After centrifugation at 5000 ×*g* for 5 min, supernatants were collected, and absorbance was measured at 260 nm using a spectrophotometer (Thermo Fisher Scientific, USA). Higher absorbance indicated increased DNA leakage, reflecting membrane disruption.

#### Determination of total extracellular protein content

Total extracellular protein release was quantified using the Bradford assay^15^, based on protein binding to Coomassie Brilliant Blue G250 dye. Unbound dye has an absorption maximum at 495 nm, which shifts to 595 nm upon protein interaction. After incubation with the dye, absorbance at 595 nm was measured and compared to a blank. The percentage increase in extracellular protein content was calculated relative to the untreated control.

#### Potassium ion (K⁺) leakage assay

Potassium ion (K⁺) leakage was measured to assess membrane disruption, following the method described by Akinrinlola et al.^16^. Bacterial suspensions were centrifuged at 5000 × g for 5 min, and the resulting supernatants were analyzed for K⁺ concentration using a flame photometer (Jenway PFP7, UK). K⁺ was expressed in ppm relative to the control.

#### Cell surface hydrophobicity assay (MATH)

Cell surface hydrophobicity was evaluated using the microbial adhesion to hydrocarbons (MATH) assay, as described by Rosenberg et al.^17^. Bacterial cells were harvested by centrifugation and resuspended in phosphate-buffered saline (PBS) to an optical density (OD₆₀₀) of approximately 1.0. Equal volumes (3 mL) of bacterial suspension and hexadecane were vortexed for 2 min and then allowed to separate for 15 min at room temperature. The aqueous phase was carefully removed, and its OD₆₀₀ was measured. Hydrophobicity (%) was calculated using the following equation:

3 $${\text{Hydrophobicity }}\left( \% \right) = \left[ {{1} - \left( {{\mathrm{OD}}_{{{\mathrm{After}}}} /{\mathrm{OD}}_{{{\mathrm{Initial}}}} } \right)} \right] \times {1}00$$

#### Virulence factors assays

*Staphyloxanthin-pyocyanin quantification* Staphyloxanthin is a golden carotenoid pigment produced by *Staphylococcus aureus*, which acts as an antioxidant. Pyocyanin is a blue phenazine pigment secreted by *Pseudomonas aeruginosa*. It is a major virulence factor that generates free radicals, damaging host tissues. Staphyloxanthin production in *Staphylococcus aureus* ATCC 25,923 was measured using a solvent extraction–spectrophotometric method, as described by Ebert et al.^18^. In brief, 5 mL of an overnight culture grown in tryptic soy broth (TSB) at 37°C was centrifuged at 6000 ×*g* for 5 min. The cell pellet was then washed twice with PBS and resuspended in 1 mL of methanol. Pigment extraction was carried out at 55°C for 5 min with intermittent vortexing. After centrifugation, the supernatant was collected, and its absorbance was measured at 465 nm. The pigment concentration was calculated using the specific extinction coefficient (4.3 × 10^3^ A₄₆₅ units per OD₆₀₀ of culture).

Pyocyanin from *Pseudomonas aeruginosa* ATCC 27,853 was quantified using the method of Essar et al*.*^19^. Bacteria were grown in King’s A medium at 37 °C with shaking (200 rpm) for 24 h. Cultures were centrifuged at 8000 ×*g* for 10 min and the supernatant was extracted with an equal volume of chloroform. The organic phase was re-extracted into 0.2 N HCl (1:1 volume), resulting in a pink aqueous phase. Absorbance was measured at 520 nm, and pyocyanin concentration was calculated as A₅₂₀ × 17.072 (µg/mL).

*Protease activity* Protease activity was assessed using casein as the substrate^20–23^. Briefly, 500 µL of bacterial supernatant was incubated at 37 °C for 30 min in a water bath with 0.2% casein (dissolved in 0.1 M NaOH) and 0.05 M phosphate buffer (pH 7). The reaction was terminated by adding 500 µL of 10% trichloroacetic acid (TCA). Subsequently, 0.5 mL of the supernatant was mixed with 2.5 mL of 0.5 M Na₂CO₃ and 0.75 mL of diluted Folin–Ciocalteu reagent (1:3, v/v with water). The mixture was incubated at room temperature in the dark for 30 min. Absorbance was recorded at 660 nm. Protease inhibition (%) was calculated using the following equation:

4 $$\% {\text{ of protease inhibition}} = \left[ {\left( {{\mathrm{OD}}_{{{\mathrm{Control}}}} - {\text{ OD}}_{{{\mathrm{Treated}}}} } \right)/{\text{ OD}}_{{{\mathrm{Control}}}} } \right] \times {1}00$$

*Esterase production* Esterase activity was assessed using a Tween 80 opacity medium^44^. A 100 μL aliquot of bacterial suspensions was inoculated into wells on nutrient agar (pH 7) composed of 0.5% peptone, 0.3% yeast extract, 0.5% NaCl, and 1.5% agar, supplemented with 0.01% CaCl₂ and 0.6% (v/v) Tween 80. Tween 80 was sterilized separately by filtration through a 0.22 μm syringe filter before addition. Plates were incubated at 37 °C for 2–4 days. Esterase production was indicated by the appearance of opaque precipitation zones surrounding bacterial colonies. The diameters of the colonies and the surrounding opaque zones were measured in millimeters. The esterase index (Pz value) was calculated using the following formula:

5 $${\mathrm{Pz}} = {\text{Bacterial colony diameter}}/\left( {{\text{Bacterial colony diameter}} + {\text{Opaque zone}} {\mathrm{diameter}}} \right)$$

*Hemolysin production* Hemolytic activity was assessed using defibrinated sheep red blood cells (RBCs). Cells were resuspended in 1X PBS pH 7.4 to achieve a 10% (v/v) solution following three rounds of washing, and then further diluted in 1X PBS (1:10). The absorbance was measured at 450 nm after 100 μl of the freshly made red blood cell suspension and 100 μl of the bacterial supernatant were combined, incubated for one hour at 37°C, then centrifuged for ten minutes at 2000 rpm (20°C). 1% TritonX-100 was used as a positive control, and the absorbance of RBCs in PBS (no lysis) was used as a negative control^24–26^.

6 $$\% {\text{ Hemolysis}} = \left[ { \, \left( {{\mathrm{A}}_{{{\mathrm{Sample}}}} - {\mathrm{A}}_{{\text{Negative Control}}} } \right)/\left( {{\mathrm{A}}_{{\text{Positive Control}}} - {\text{ A}}_{{\text{Negative Control}}} } \right)} \right] \times {1}00$$

*Biofilm formation assay* Biofilm formation was quantified using the crystal violet staining method in 96-well microtiter plates, as described by O’Toole^27^. Bacterial suspensions (200 µL, 10⁸ CFU/mL) were inoculated into sterile wells and incubated at 37 °C for 24 h. After incubation, wells were gently washed three times with PBS to remove planktonic cells and stained with 0.1% crystal violet for 15 min. Excess dye was removed, and the wells were rinsed and air-dried. Bound dye was solubilized in 33% acetic acid, and absorbance was measured at 570 nm using a microplate reader (BioTek, USA).

### Ultrastructure changes determination by SEM

SEM was used to visualize the morphological changes in the two bacterial strains under investigation under various treatments. Initially, bacterial cells were fixed for 12 h using 3% glutaraldehyde in phosphate buffer saline (PBS). The bacterial cultures were then dehydrated in an ethanol gradient (25%, 50%, 75%, and 100%) after being cleaned for two hours with 4% OsO4 in 0.1 M phosphate buffer. Following the gold coating process, the samples were analyzed using a SEM (JEOL JSM 6360LA, Japan).

### Statistical analysis of the data

All quantitative data are presented as mean ± standard deviation (SD) from three independent biological replicates (n = 3). Data were analyzed using IBM SPSS Statistics version 25.0 (IBM Corp., USA). One-way analysis of variance (ANOVA) was used to determine significant differences among treatment groups. Data normality and homogeneity of variances were verified using the Shapiro–Wilk and Levene’s tests, respectively. Post-hoc pairwise comparisons were performed using Tukey’s Honestly Significant Difference (HSD) test at a confidence level of 95% (α = 0.05).

Bar charts were generated in Microsoft Excel 2023 for visual presentation. Figures include error bars representing the standard deviation (SD), and statistically significant differences between groups (p < 0.05) are denoted by different letters above the bars.

## Results

### Silver nanoparticle characterization

Because in case of nanoparticles, the released ions are not the only factor, but also the physicochemical characteristics of them are extremely important, the physicochemical characteristics of the purchased silver nanoparticles (Ag-NPs) were evaluated using various analytical techniques.

#### Morphological analysis

**Fig. 1:**
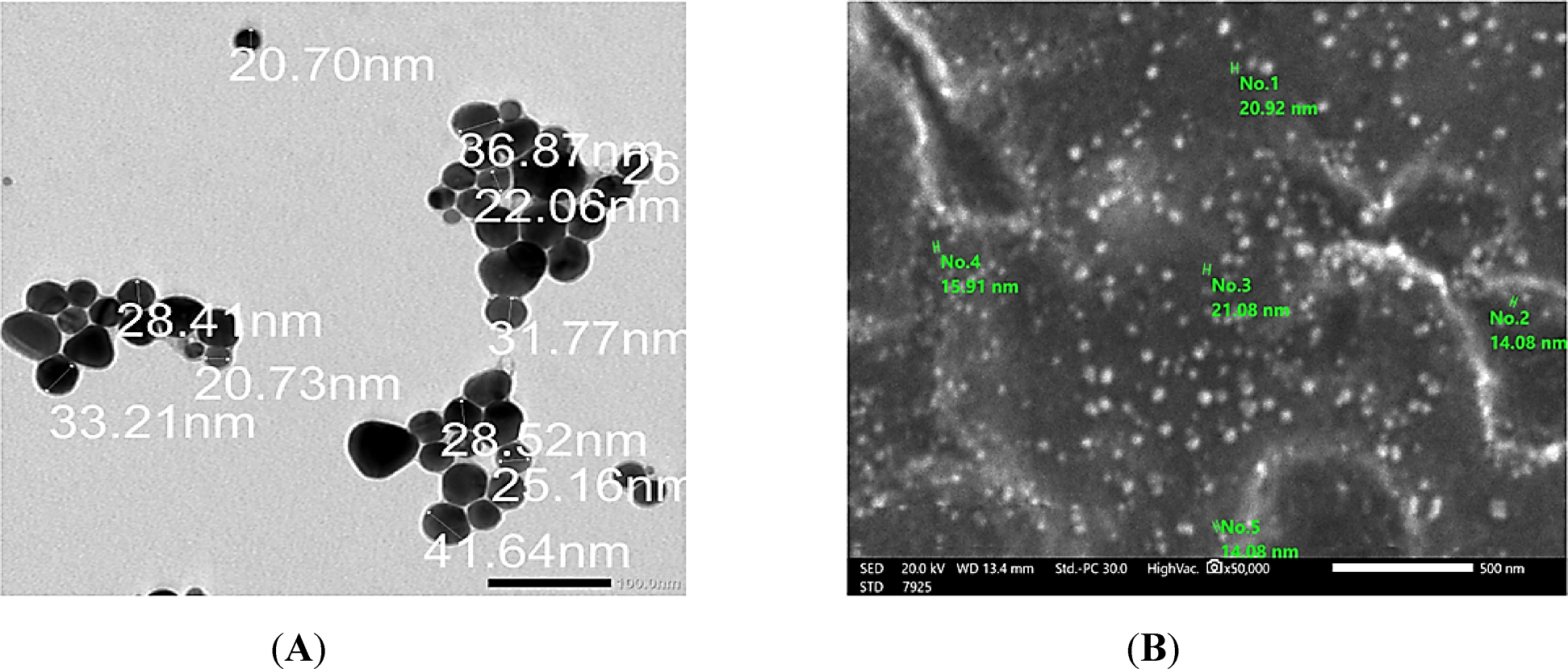
Morphological analysis of Ag-NPs : (**A**) SEM Image; (**B**) TEM Image.

 The surface morphology and particle size of the Ag-NPs were examined using scanning electron microscopy (SEM) and transmission electron microscopy (TEM) (Fig. 1 A and B). The SEM image revealed a relatively uniform distribution of spherical particles with minimal agglomeration. TEM analysis confirmed the spherical morphology and showed well-dispersed nanoparticles with a mean particle diameter of 28.9 ± 7 nm.

#### Optical properties

**Fig. 2:**
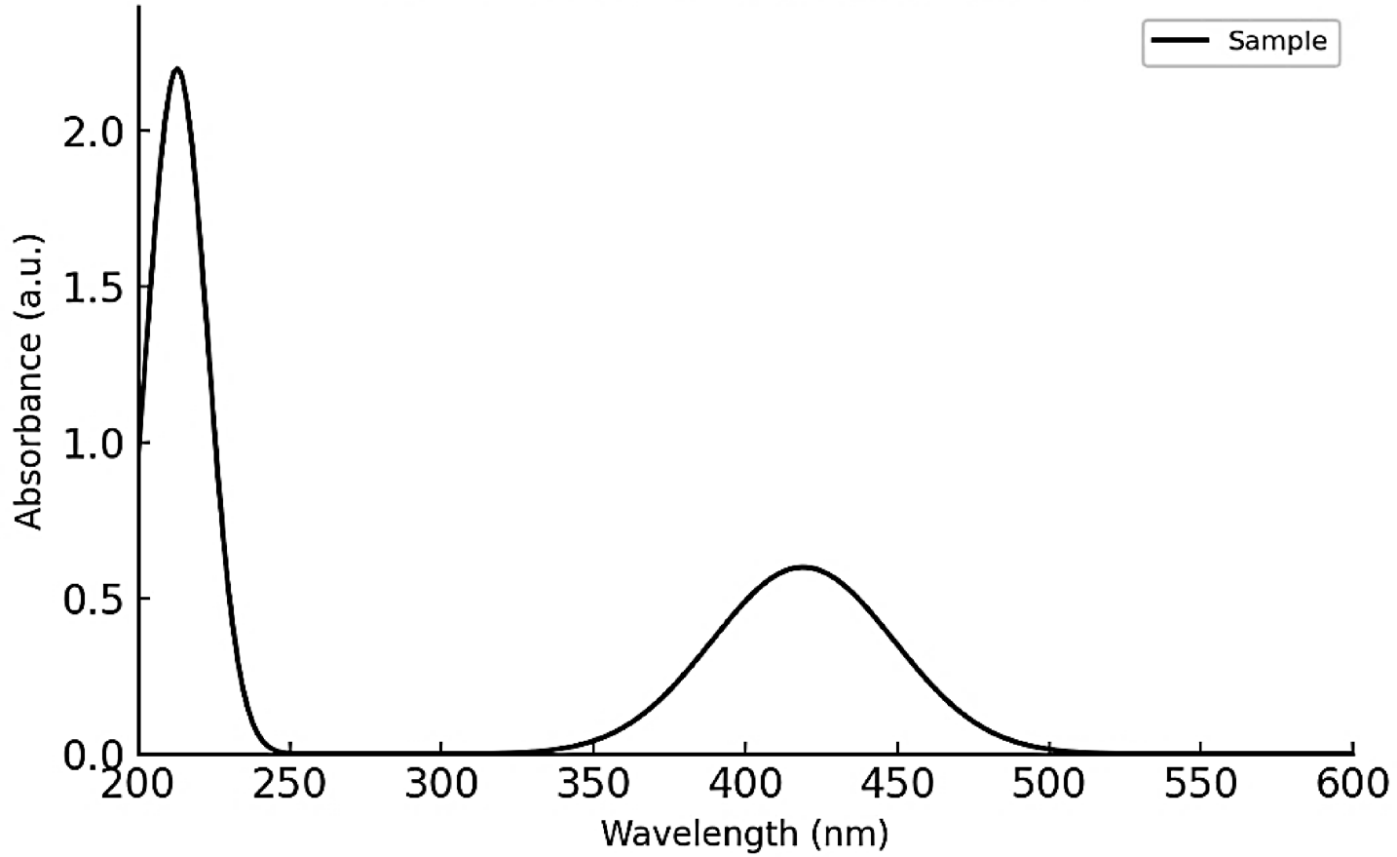
UV–Vis spectrum of Ag-NPs.

The UV–Vis absorption spectrum of the Ag-NPs exhibited a strong surface plasmon resonance (SPR) band centered at approximately 419 nm (Fig. 2), which is characteristic of well-dispersed spherical silver nanoparticles. The presence of this distinct SPR peak indicates the collective oscillation of conduction electrons in response to incident light, a typical feature of metallic nanostructures. The relatively sharp and symmetric nature of the peak suggests a narrow size distribution and good colloidal stability. Additionally, the absence of secondary peaks or broad absorption in the visible range confirms minimal aggregation and the formation of predominantly monodisperse nanoparticles.

#### Zeta potential measurement

**Fig. 3:**
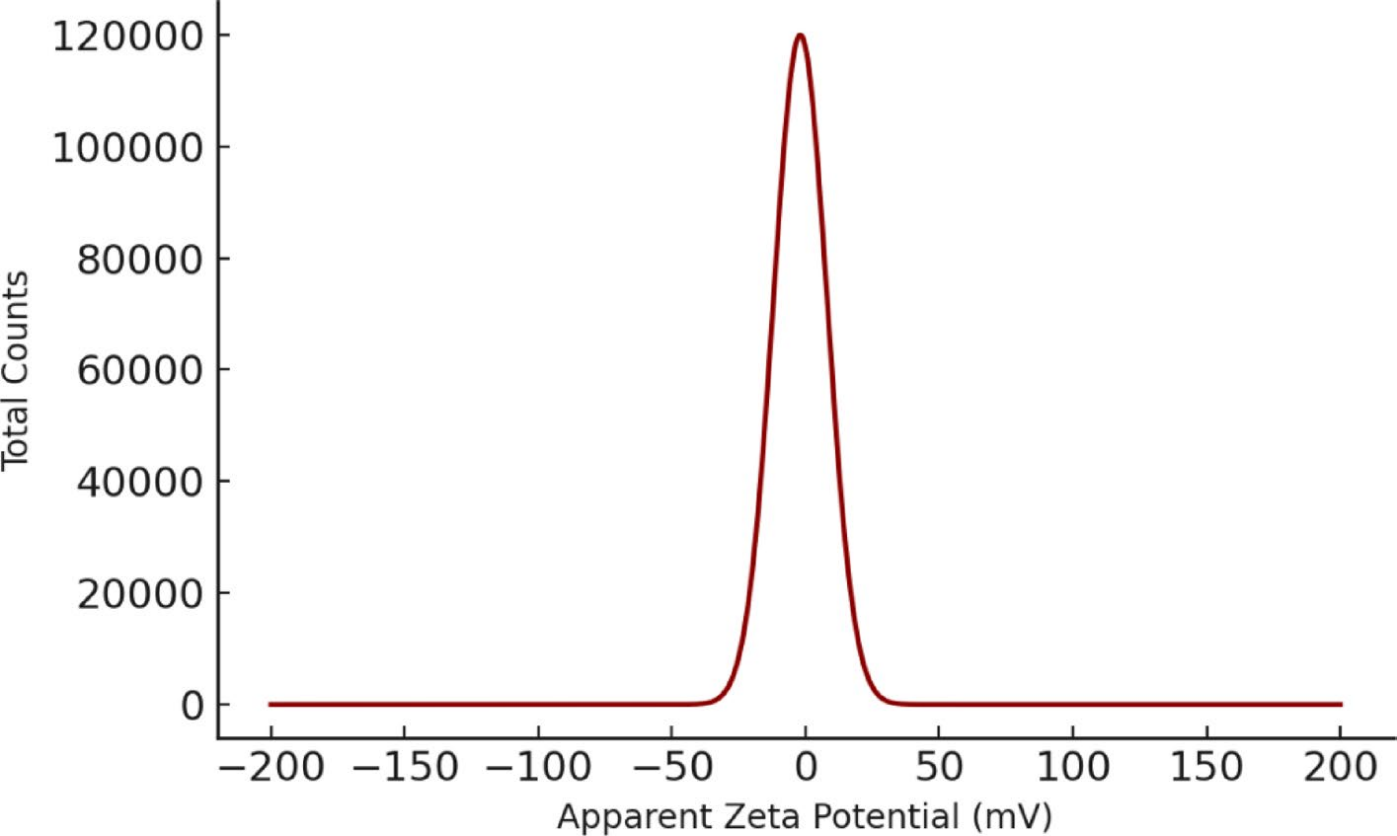
Zeta potential distribution of Ag-NPs.

The surface charge and colloidal stability of the Ag-NPs were assessed via zeta potential measurements. The particles exhibited a zeta potential of − 14.4 mV (Fig. 3), indicating moderate stability in aqueous suspension. A negative zeta potential of this magnitude suggests that the nanoparticles are stabilized primarily through electrostatic repulsion, which reduces the likelihood of aggregation. This value is consistent with partially capped silver nanoparticles dispersed in water without strong surfactant stabilization.

#### Crystallographic structure

**Fig. 4:**
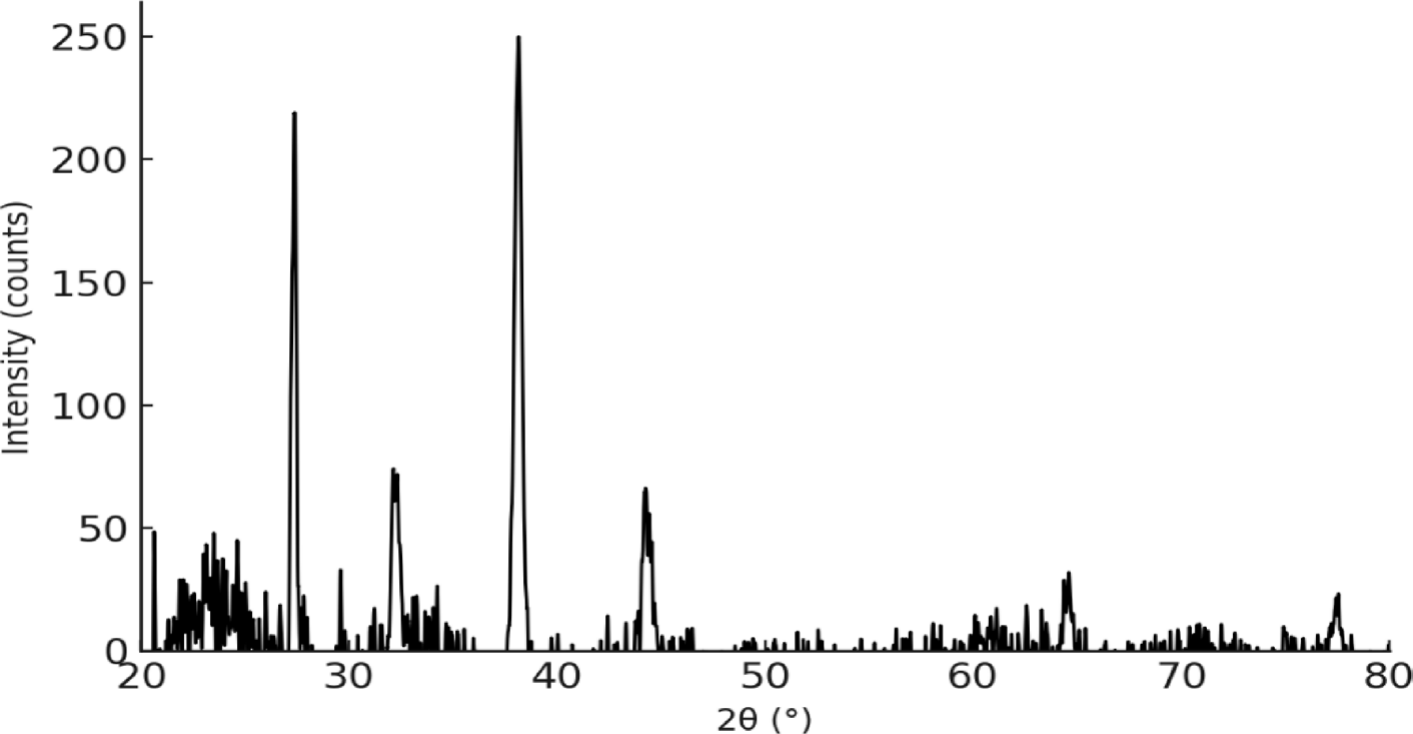
XRD pattern of Ag-NPs.

The crystalline structure of the Ag-NPs was analyzed by X-ray diffraction (XRD) (Fig. 4). The diffraction pattern revealed distinct peaks at 2θ values of approximately 38.1°, 44.3°, and 64.4°, which correspond to the (111), (200), and (220) crystal planes of face-centered cubic (fcc) silver, in agreement with the standard reference (JCPDS No. 04–0783). The presence of sharp and intense peaks indicates a high degree of crystallinity, confirming the successful formation of metallic silver nanoparticles. No additional peaks from impurities or secondary phases were observed, suggesting the high purity of the purchased Ag-NPs.

#### Functional group analysis

**Fig. 5:**
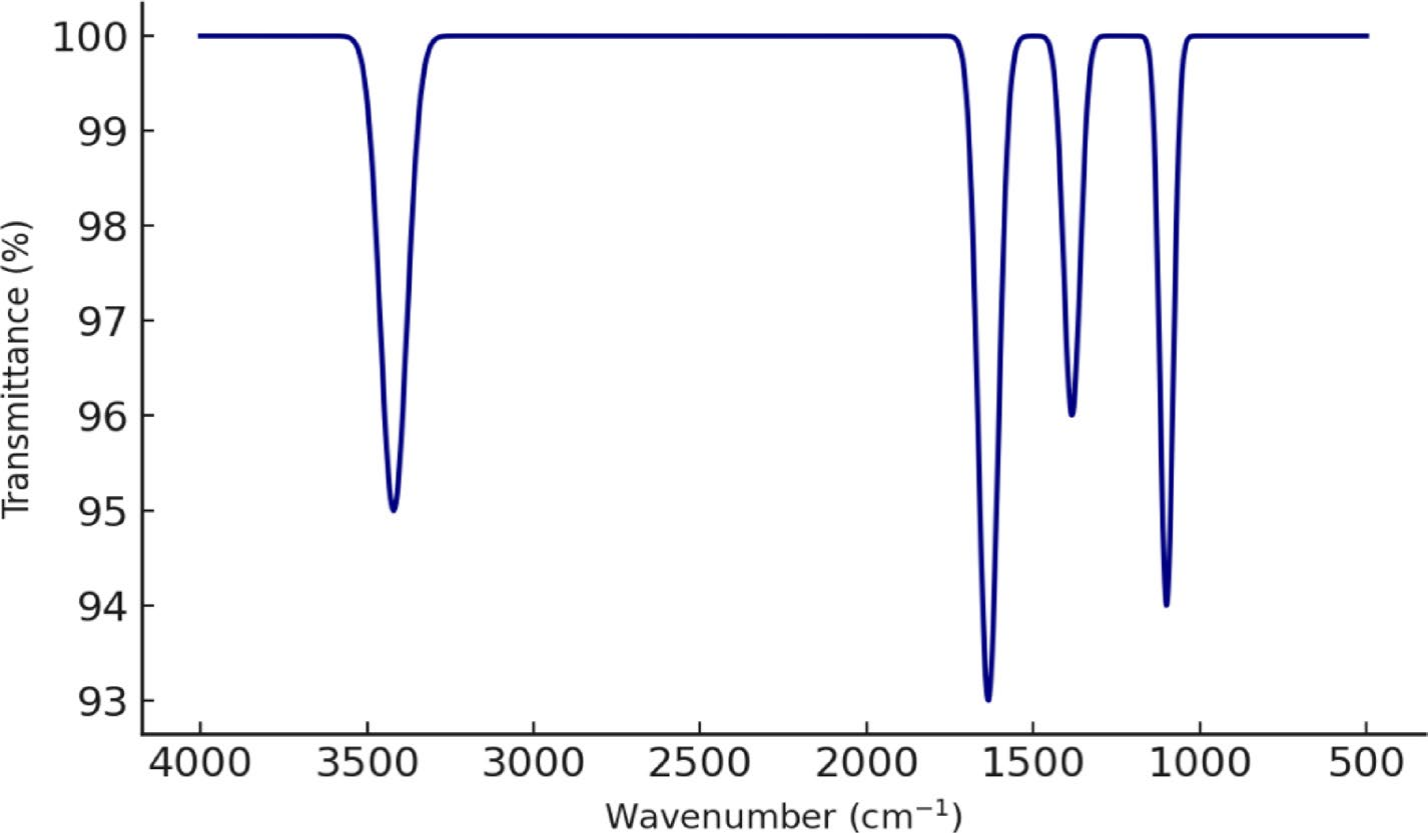
FTIR spectrum of Ag-NPs.

FTIR analysis (Fig. 5) was conducted to identify the surface functional groups present on the Ag-NPs. The spectrum displayed prominent absorption bands at 3420 cm⁻^1^, 1635 cm⁻^1^, 1384 cm⁻^1^, and 1100 cm⁻^1^. The broad peak at 3420 cm⁻^1^ corresponds to O–H or N–H stretching vibrations, indicating the presence of hydroxyl or amine groups. The strong band at 1635 cm⁻^1^ is attributed to C = O stretching, likely from carboxylic or amide functionalities. The peak at 1384 cm⁻^1^ is characteristic of symmetric NO₃⁻ stretching, while the absorption near 1100 cm⁻^1^ is associated with C–O stretching vibrations. These functional groups suggest the involvement of organic molecules, possibly residual stabilizers or capping agents, which enhance the colloidal stability of Ag-NPs in aqueous media.

### Silver ion concentration analysis

The concentration of silver ions (Ag +) in the samples generated by electrolysis, chemical dissolution (AgNO₃), and Ag-NPs was quantified using ICP-OES, and the results are summarized in Table 1.

**Table 1:** Soluble silver concentration of the tested silver sources. Silver concentrations are expressed as mg/L (ppm), representing the soluble fraction (mainly Ag⁺ ions) measured by ICP-OES after 0.2 µm filtration.

Source	Condition	Ag + Concentration
DC electrolysis	5 V—10 min	9.51 ± 0.50
DC electrolysis	5 V—20 min	18.92 ± 1.00
DC electrolysis	5 V—30 min	23.25 ± 1.20
DC electrolysis	10 V—10 min	76.39 ± 3.00
DC electrolysis	10 V—20 min	117.59 ± 4.50
DC electrolysis	10 V—30 min	214.09 ± 6.00
DC electrolysis	15 V—10 min	145.20 ± 5.00
DC electrolysis	15 V—20 min	278.90 ± 7.00
DC electrolysis	15 V—30 min	412.49 ± 8.00
Ag-NPs suspension	1.00 mL	1513.3 ± 30.3
Ag-NPs suspension	0.50 mL	652.5 ± 15.0
Ag-NPs suspension	0.25 mL	311.4 ± 7.5
AgNO₃ solution	1.00 g/mL	806,314 ± 12,095
AgNO₃ solution	0.50 g/mL	395,094 ± 7,902
AgNO₃ solution	0.25 g/mL	190,495 ± 4,762

Electrolysis at increasing voltages and longer durations resulted in progressively higher Ag + release. At 5 V, the Ag + concentration rose from 9.51 ppm at 10 min to 18.92 ppm at 20 min and 23.25 ppm at 30 min. Similarly, 10 V produced 76.39 ppm, 117.59 ppm, and 214.09 ppm at the same time intervals. The most pronounced effect was observed at 15 V, where concentrations increased from 145.20 ppm (10 min) to 278.90 ppm (20 min) and 412.49 ppm (30 min). These results demonstrate that both voltage and exposure time are positively correlated with the ionization rate of silver electrodes. In the case of Ag-NPs, the Ag + concentration scaled proportionally with the volume of suspension tested, yielding 311.4 ppm for 0.25 mL, 652.5 ppm for 0.5 mL, and 1513.3 ppm for 1 mL. This reflects a consistent and dose-dependent release of silver ions from the colloidal nanoparticle sample. Silver nitrate (AgNO₃) solutions exhibited the highest ionic concentrations, yielding 190,495 ppm (0.25 g), 395,094 ppm (0.5 g), and 806,314 ppm (1 g) when dissolved in 1 mL of water. These extremely high values confirm the full dissociation of silver nitrate in aqueous media, making it the most concentrated ionic source of silver among the tested methods.

Based on these results, and to ensure a fair comparison of antibacterial potency, an equivalent silver ion (Ag⁺) concentration of approximately 400 ppm was standardized across all treatments. This concentration was achieved through three distinct sources: (1) electrolyzed Ag + , generated by DC application of 15 V for 30 min during DC electrolysis; (2) Ag-NPs Ag + , using 0.3 mL of Ag-NPs suspension; and (3) AgNO₃ Ag + yielding from silver nitrate, by diluting a 0.5 g/mL solution by 1000-fold.

### The effect of different exposure conditions on examined pathogens

#### Cultivability assessment

By counting them both before and after exposure, the inhibitory effectiveness of Ag^+^ applied methods was examined against *P. aeruginosa* and *S. aureus*; the results are displayed in Table 2. Exposure of *P. aeruginosa* and *S. aureus* to silver ions from three sources resulted in marked reductions in viable bacterial counts compared to the untreated control. For *P. aeruginosa*, electrolyzed silver ions produced the highest inhibitory effect, reducing CFU from 1.95 ± 0.09 × 10^1^⁰/mL in the control to 0.17 ± 0.08 × 10^1^⁰/mL (p < 0.001), corresponding to 91.06% inhibition. Ag-NPs and AgNO₃ showed moderate reductions (81.87% and 75.32%, respectively), with no statistically significant difference between them.

**Table 2:** Effect of silver ion sources on bacterial viability of *P. aeruginosa* and *S. aureus*.

Treatment	*P. aeruginosa* (CFU × 101^ο^/mL)	Inhibition (%)	Treatment	*S. aureus* (CFU × 101^ο^/mL)	Inhibition (%)
Control	1.95 ± 0.09 *	0.00	Control	1.62 ± 0.2 *	0.00
Electrolyzed Ag +	0.17 ± 0.08 **	91.06	Electrolyzed Ag +	0.47 ± 0.09 **	71.10
Ag-NPs Ag +	0.35 ± 0.04	81.87	Ag-NPs Ag +	0.66 ± 0.11	59.4
AgNO₃ Ag +	0.48 ± 0.02	75.32	AgNO₃ Ag +	0.75 ± 0.08	53.8

* Significant differences were found between the control and the other treatments (p < 0.01). ** Significant differences were found between electrolyzed Ag⁺ and the other treatments (p < 0.01).

Similarly, in *S. aureus*, the electrolyzed silver ions again achieved the most pronounced effect, reducing the CFU to 0.47 ± 0.09 × 10^1^⁰/mL (p < 0.001), which equates to 71.10% inhibition. Ag-NPs and AgNO₃ treatments resulted in 59.4% and 53.8% inhibition, respectively, again without a significant difference between them.

There was no significant difference between Ag-NPs and AgNO₃ (p > 0.05).

#### Metabolic activity

**Fig. 6:**
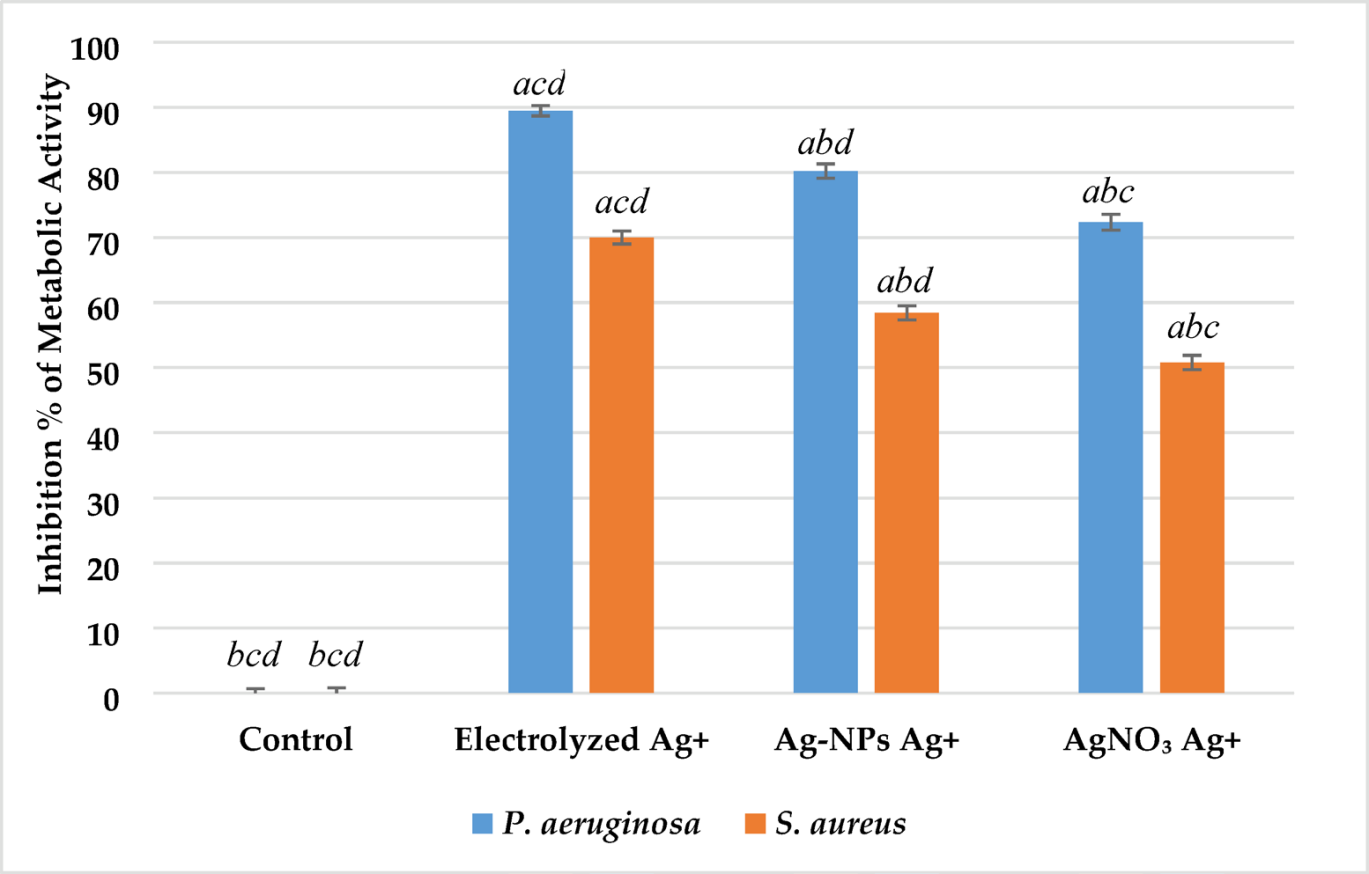
Effect of silver ion sources on the metabolic activity inhibition percentage of *P. aeruginosa* and *S. aureus*. Data represent mean ± SD (n = 3). Statistical significance was determined by one-way ANOVA followed by Tukey’s HSD post-hoc test (p < 0.05). Different letters above the bars indicate significant differences among treatments: *a*—Control, *b*—Electrolyzed Ag⁺, *c*—Ag-NPs Ag⁺, and *d*—AgNO₃ Ag⁺.

The metabolic activity of *P. aeruginosa* and *S. aureus* was significantly suppressed (p < 0.05) with silver ions from all sources (Fig. 6). Electrolyzed silver ions demonstrated the highest inhibition rates, with *P. aeruginosa* showing 89.5 ± 3.7% inhibition and *S. aureus* 70.0 ± 4.2% (p < 0.001). Ag-NPs also produced notable inhibition, reaching 80.2 ± 5.1% for *P. aeruginosa* and 58.4 ± 3.8% for *S. aureus* (p < 0.01). The lowest inhibition was observed in the AgNO₃ treatment group, with 72.3 ± 3.5% for *P. aeruginosa* and 50.8 ± 4.5% for *S. aureus* (p < 0.05). These results follow the same trend observed in cultivability assays, confirming that electrolyzed Ag⁺ ions possess the most potent antibacterial metabolic impact, followed by Ag-NPs and finally AgNO₃. The greater suppression observed in *P. aeruginosa* compared to *S. aureus* may reflect species-specific susceptibility to silver-based antimicrobial agents.

#### Sliding motility

**Fig. 7:**
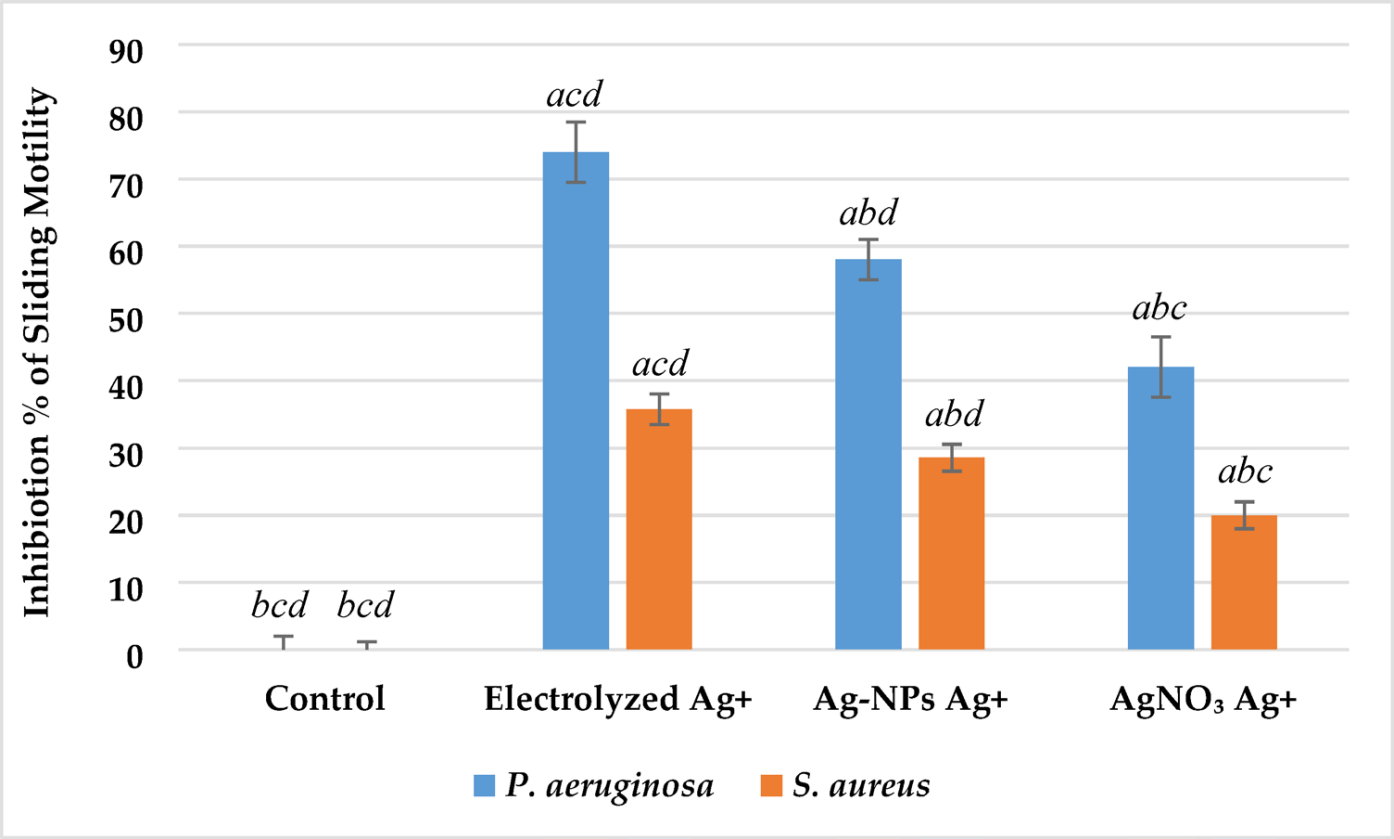
Effect of silver ion sources on the sliding motility inhibition percentage of *P. aeruginosa* and *S. aureus*. Data represent mean ± SD (n = 3). Statistical significance was determined by one-way ANOVA followed by Tukey’s HSD post-hoc test (p < 0.05). Different letters above the bars indicate significant differences among treatments: *a*—Control, *b*—Electrolyzed Ag⁺, *c*—Ag-NPs Ag⁺, and *d*—AgNO₃ Ag⁺.

The inhibitory effects of various Ag⁺ sources on the sliding motility of *P. aeruginosa* and *S. aureus* are shown in Fig. 7. The results indicated that all tested forms of Ag⁺ significantly reduced sliding motility compared to the untreated control (p < 0.05), with notable differences in potency among the sources. For *P. aeruginosa*, sliding motility decreased from 5.0 ± 0.3 mm in the control to 1.3 ± 0.1 mm after treatment with electrolyzed Ag⁺ , representing a 74% inhibition. Treatments with Ag-NPs and AgNO₃ caused inhibition levels of 58% and 42%, with residual motility diameters of 2.1 ± 0.15 mm and 2.9 ± 0.2 mm, respectively. Similarly, *S. aureus* showed reduced sliding motility from 7.0 ± 0.55 mm in the control to 4.5 ± 0.35 mm (35% inhibition) following electrolyzed Ag⁺ treatment. Treatments with Ag-NPs and AgNO₃ resulted in less pronounced effects, with motility diameters of 5.0 ± 0.45 mm (29% inhibition) and 5.6 ± 0.55 mm (20% inhibition), respectively.

#### DNA leakage

**Fig. 8:**
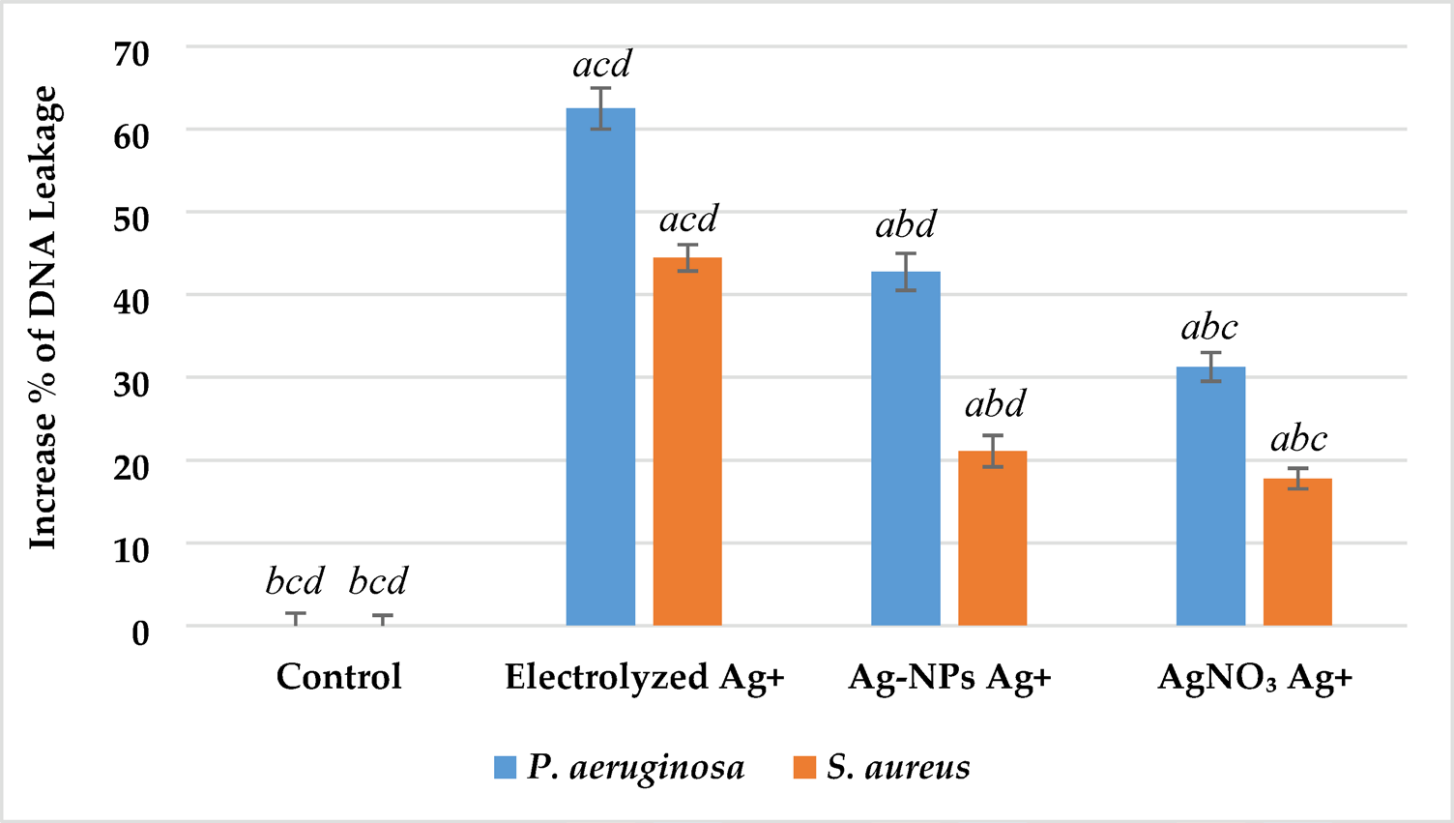
Effect of Silver Ion Sources on the DNA Leakage Increase Percentage of *P. aeruginosa* and *S. aureus*. Data represent mean ± SD (n = 3). Statistical significance was determined by one-way ANOVA followed by Tukey’s HSD post-hoc test (p < 0.05). Different letters above the bars indicate significant differences among treatments: *a*—Control, *b*—Electrolyzed Ag⁺, *c*—Ag-NPs Ag⁺, and *d*—AgNO₃ Ag⁺.

DNA leakage was quantified by measuring absorbance at 260 nm, indicating disruption of cell membrane integrity. As shown in Fig. 8, all silver treatments significantly increased DNA release compared to the untreated control (p < 0.05). In *P. aeruginosa*, DNA leakage increased by 63% ± 2.8 with electrolyzed Ag⁺, 44% ± 2.1 with Ag-NPs Ag⁺, and 32% ± 1.9 with AgNO₃ Ag⁺. For *S. aureus*, increases were 45% ± 2.3, 23% ± 1.5, and 18% ± 1.2, respectively. In both strains, electrolyzed Ag⁺ induced significantly greater DNA leakage than Ag-NPs or AgNO₃ (p < 0.05).

#### Total extracellular protein content

**Fig. 9:**
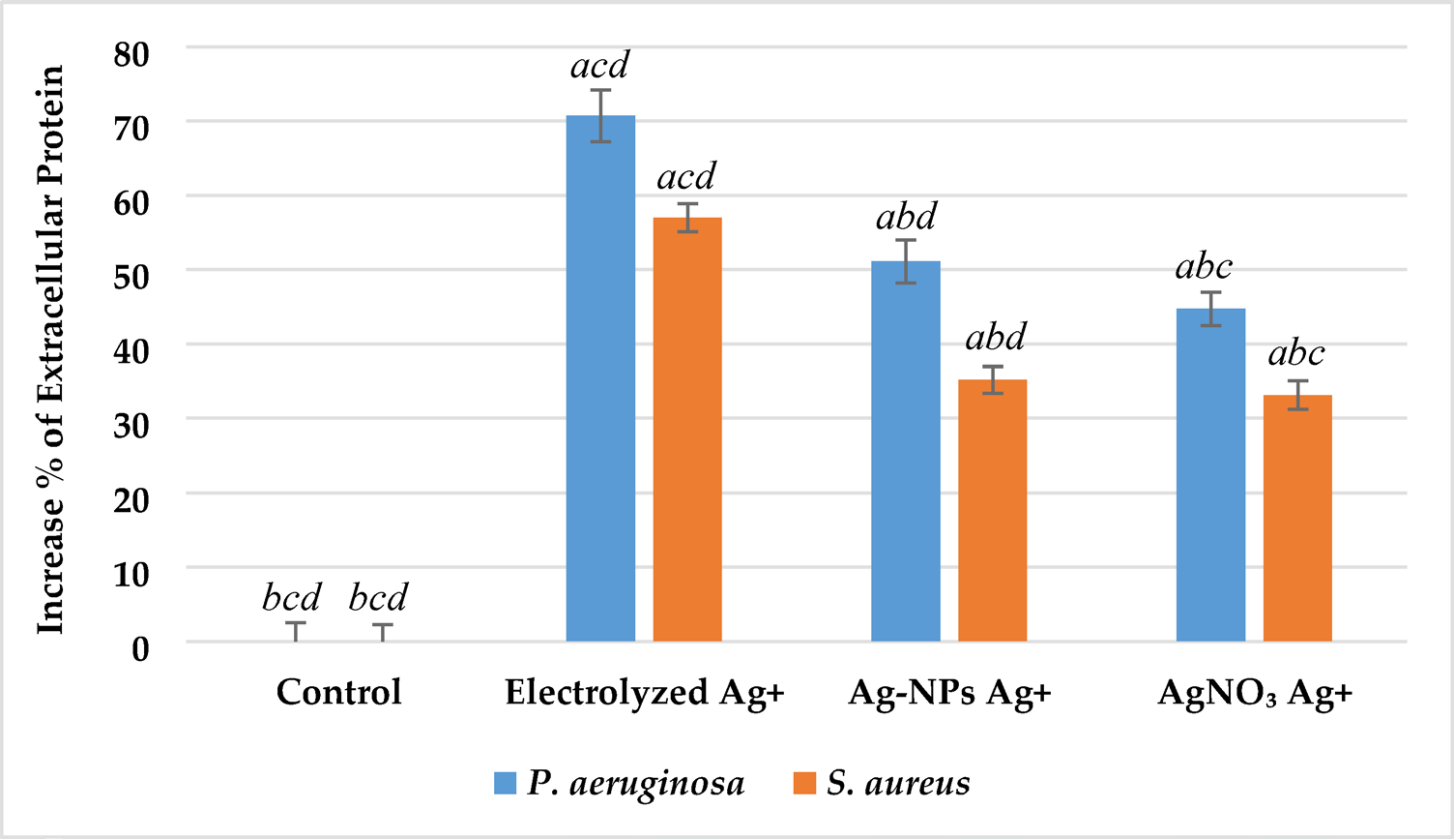
Effect of silver ion sources on the total extracellular protein increase percentage of *P. aeruginosa* and *S. aureus*. Data represent mean ± SD (n = 3). Statistical significance was determined by one-way ANOVA followed by Tukey’s HSD post-hoc test (p < 0.05). Different letters above the bars indicate significant differences among treatments: *a*—Control, *b*—Electrolyzed Ag⁺, *c*—Ag-NPs Ag⁺, and *d*—AgNO₃ Ag⁺.

Protein leakage was measured to further assess membrane damage. As illustrated in Fig. 9, all silver ion treatments caused significantly higher extracellular protein levels compared to the control (p < 0.05). In *P. aeruginosa*, protein leakage increased by 71% ± 3.1 with electrolyzed Ag⁺, 51% ± 2.4 with Ag-NPs Ag⁺, and 44% ± 2.0 with AgNO₃ Ag⁺. Corresponding increases for *S. aureus* were 57% ± 2.7, 37% ± 2.2, and 32% ± 1.9, respectively. Electrolyzed Ag⁺ showed significantly higher protein leakage compared to the other silver sources in both bacterial strains (p < 0.05).

#### Potassium ion (K +) ion leakage

**Fig. 10:**
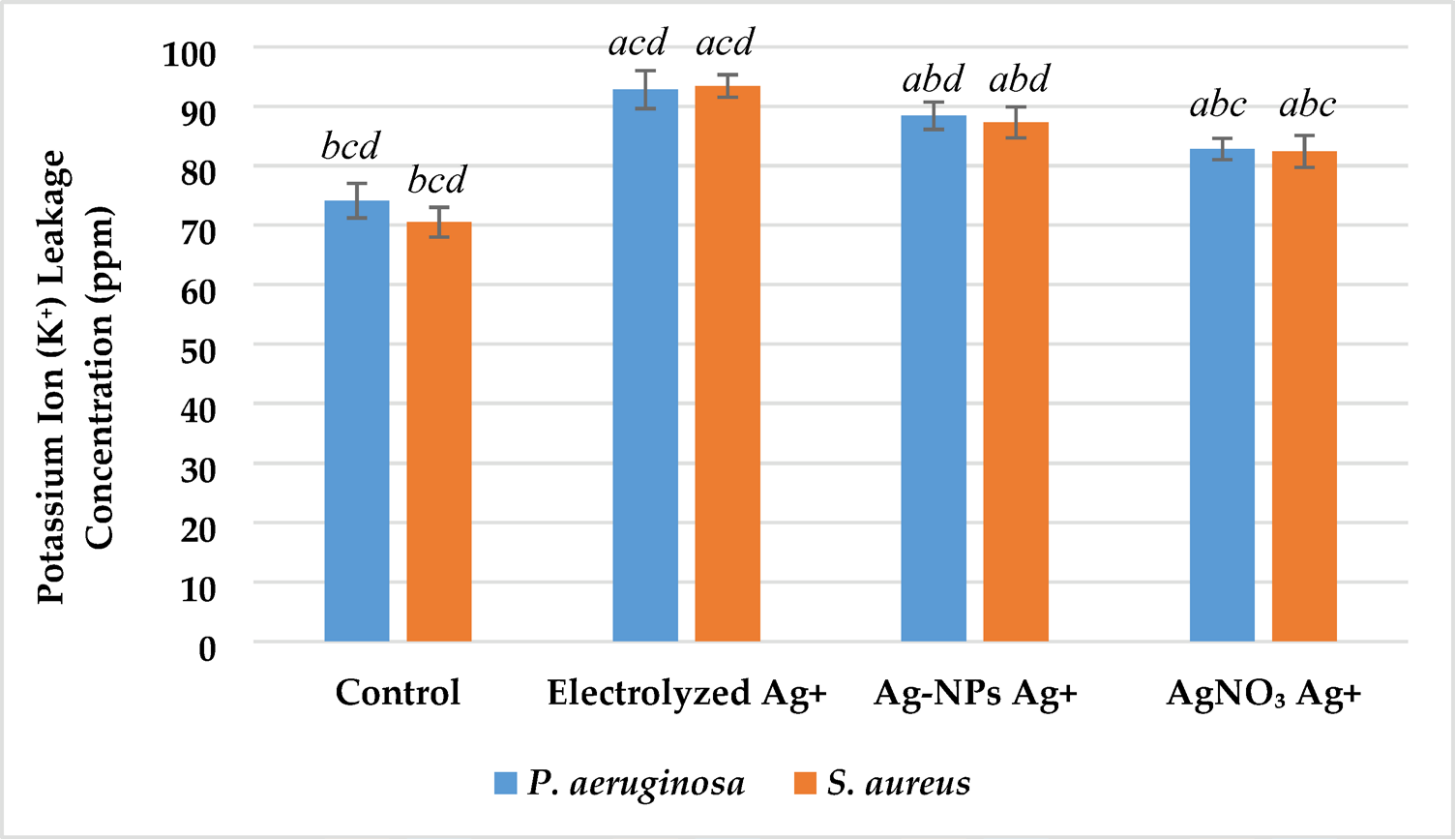
Effect of silver ion sources on the potassium ion leakage concentration (ppm) of *P. aeruginosa* and *S. aureus*. Data represent mean ± SD (n = 3). Statistical significance was determined by one-way ANOVA followed by Tukey’s HSD post-hoc test (p < 0.05). Different letters above the bars indicate significant differences among treatments: *a*—Control, *b*—Electrolyzed Ag⁺, *c*—Ag-NPs Ag⁺, and *d*—AgNO₃ Ag⁺.

Potassium ion leakage was measured as an indicator of membrane permeability disruption following treatment with different silver ion sources. As illustrated in Fig. 10, all treated groups exhibited significantly increased extracellular K⁺ concentrations compared to the untreated control (p < 0.05). In *P. aeruginosa*, K⁺ levels rose from 75.3 ± 1.9 ppm in the control to 93.5 ± 2.0 ppm with electrolyzed Ag⁺ (24.2% increase), 88.6 ± 1.8 ppm with Ag-NPs Ag⁺ (17.6%), and 82.4 ± 1.6 ppm with AgNO₃ Ag⁺ (9.4%). In *S. aureus*, concentrations increased from 72.1 ± 1.8 ppm in the control to 92.6 ± 2.1 ppm with electrolyzed Ag⁺ (28.4%), 87.9 ± 1.7 ppm with Ag-NPs Ag⁺ (21.9%), and 81.2 ± 1.5 ppm with AgNO₃ Ag⁺ (12.6%). Electrolyzed Ag⁺ induced significantly greater K⁺ leakage than both Ag-NPs and AgNO₃ treatments (p < 0.05), while all treatments were significantly different from the control in both bacterial strains.

#### Cell surface hydrophobicity

**Fig. 11:**
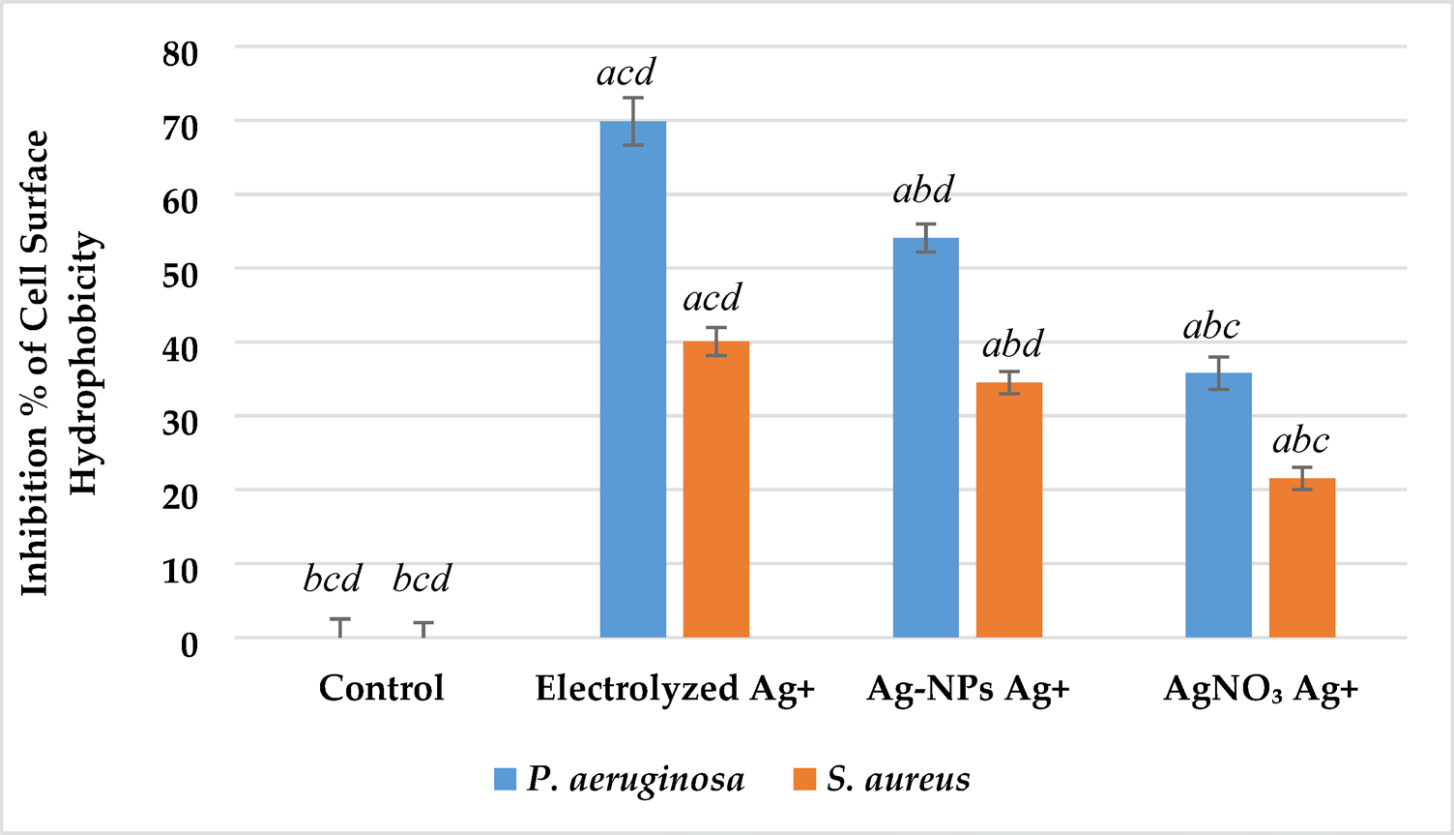
Effect of silver ion sources on the cell surface hydrophobicity inhibition percentage of *P. aeruginosa* and *S. aureus*. Data represent mean ± SD (n = 3). Statistical significance was determined by one-way ANOVA followed by Tukey’s HSD post-hoc test (p < 0.05). Different letters above the bars indicate significant differences among treatments: *a*—Control, *b*—Electrolyzed Ag⁺, *c*—Ag-NPs Ag⁺, and *d*—AgNO₃ Ag⁺.

Cell surface hydrophobicity was evaluated to assess alterations in bacterial adhesion potential following treatment with silver ions. As shown in Fig. 11, all silver-based treatments significantly reduced CSH in both *P. aeruginosa* and *S. aureus* compared to the control group (p < 0.05). In *P. aeruginosa*, the highest inhibition was observed with electrolyzed Ag⁺, reaching 69%, followed by Ag-NPs Ag⁺ (54%) and AgNO₃ Ag⁺ (33%). In *S. aureus*, a similar trend was observed with inhibition values of 41%, 34%, and 22% for electrolyzed Ag⁺, Ag-NPs Ag⁺, and AgNO₃ Ag⁺, respectively. All treatments differed significantly from the control (p < 0.05), and inhibition by electrolyzed Ag⁺ was significantly greater than that by Ag-NPs and AgNO₃ (p < 0.05).

#### Impact on bacterial virulence factors

To assess the anti-virulence potential of the tested silver ion sources, their effects on multiple bacterial virulence determinants were investigated. These included pigment production (staphyloxanthin in *S. aureus* and pyocyanin in *P. aeruginosa*), as well as extracellular enzymatic and structural factors such as protease, esterase, hemolysin activity, and biofilm formation. These virulence traits contribute to bacterial adhesion, immune evasion, host tissue damage, and chronic infection. The results of each assay are detailed below for both *P. aeruginosa* and *S. aureus*.

**Fig. 12:**
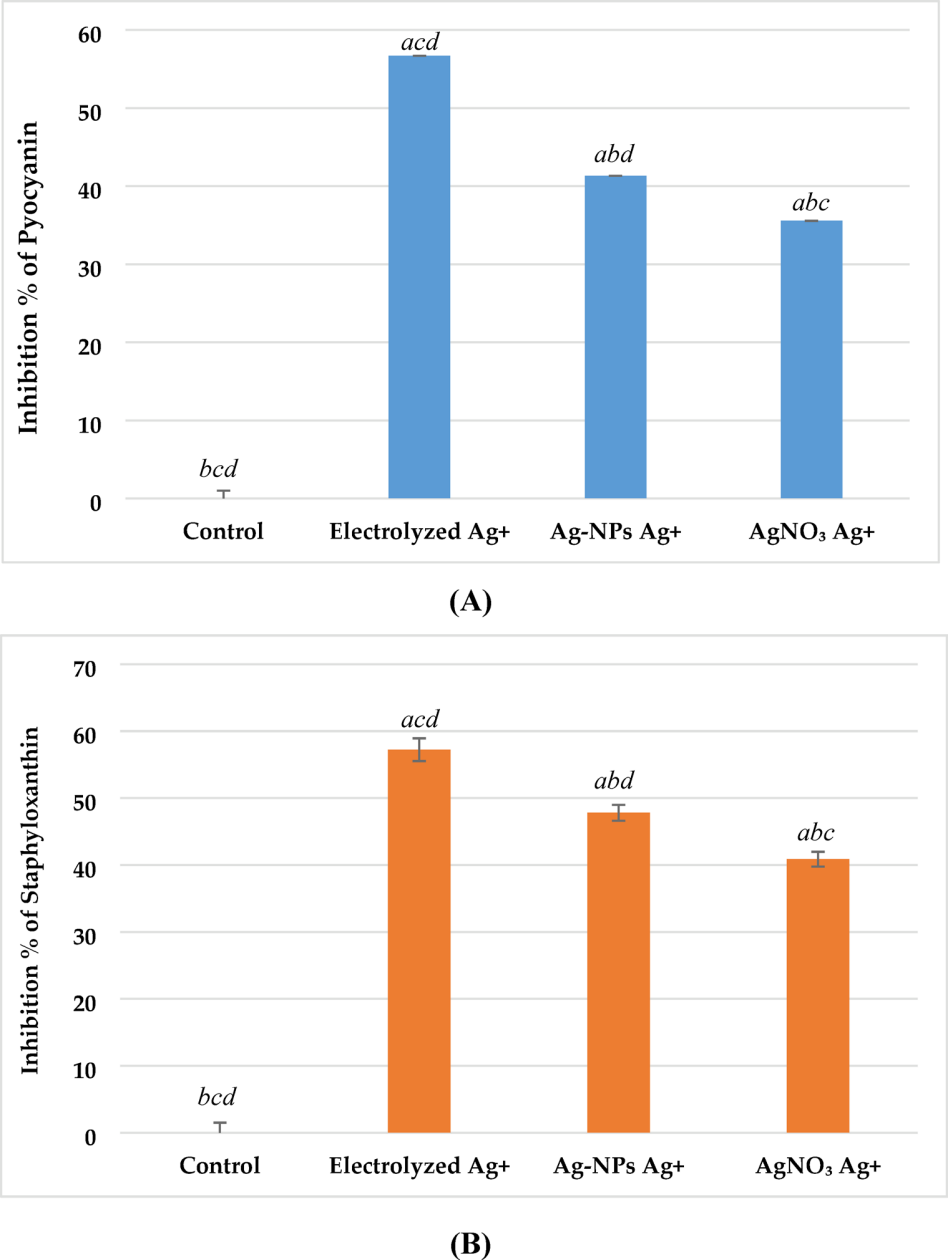
Effect of silver ion sources on the inhibition percentage of pigment production. **(A):** Inhibition of Pyocyanin Production in *P. aeruginosa*. **(B):** Inhibition of Staphyloxanthin Production in *S. aureus*. Data represent mean ± SD (n = 3). Statistical significance was determined by one-way ANOVA followed by Tukey’s HSD post-hoc test (p < 0.05). Different letters above the bars indicate significant differences among treatments: *a*—Control, *b*—Electrolyzed Ag⁺, *c*—Ag-NPs Ag⁺, and *d*—AgNO₃ Ag⁺.

*Impact on pigment production (Staphyloxanthin and Pyocyanin)* The effects of different silver ion sources on bacterial pigment production were evaluated as markers of virulence attenuation. As shown in Fig. 12, all treatments significantly reduced pigment synthesis compared to the untreated control (p < 0.001). In *P. aeruginosa*, pyocyanin production (Fig. 12A) was inhibited by 56.7 ± 2.3% with electrolyzed Ag⁺, followed by 41.3 ± 1.6% with Ag-NPs Ag⁺, and 35.6 ± 1.4% with AgNO₃ Ag⁺. In *S. aureus*, staphyloxanthin production (Fig. 12B) was similarly suppressed, showing 57.2 ± 1.7%, 47.8 ± 1.2%, and 40.9 ± 1.1% inhibition with electrolyzed Ag⁺, Ag-NPs Ag⁺, and AgNO₃ Ag⁺, respectively. Electrolyzed Ag⁺ resulted in significantly greater inhibition of pigment production in both species when compared to Ag-NPs and AgNO₃ (p < 0.01), indicating a stronger impact on pigment-regulated virulence mechanisms.

**Fig. 13:**
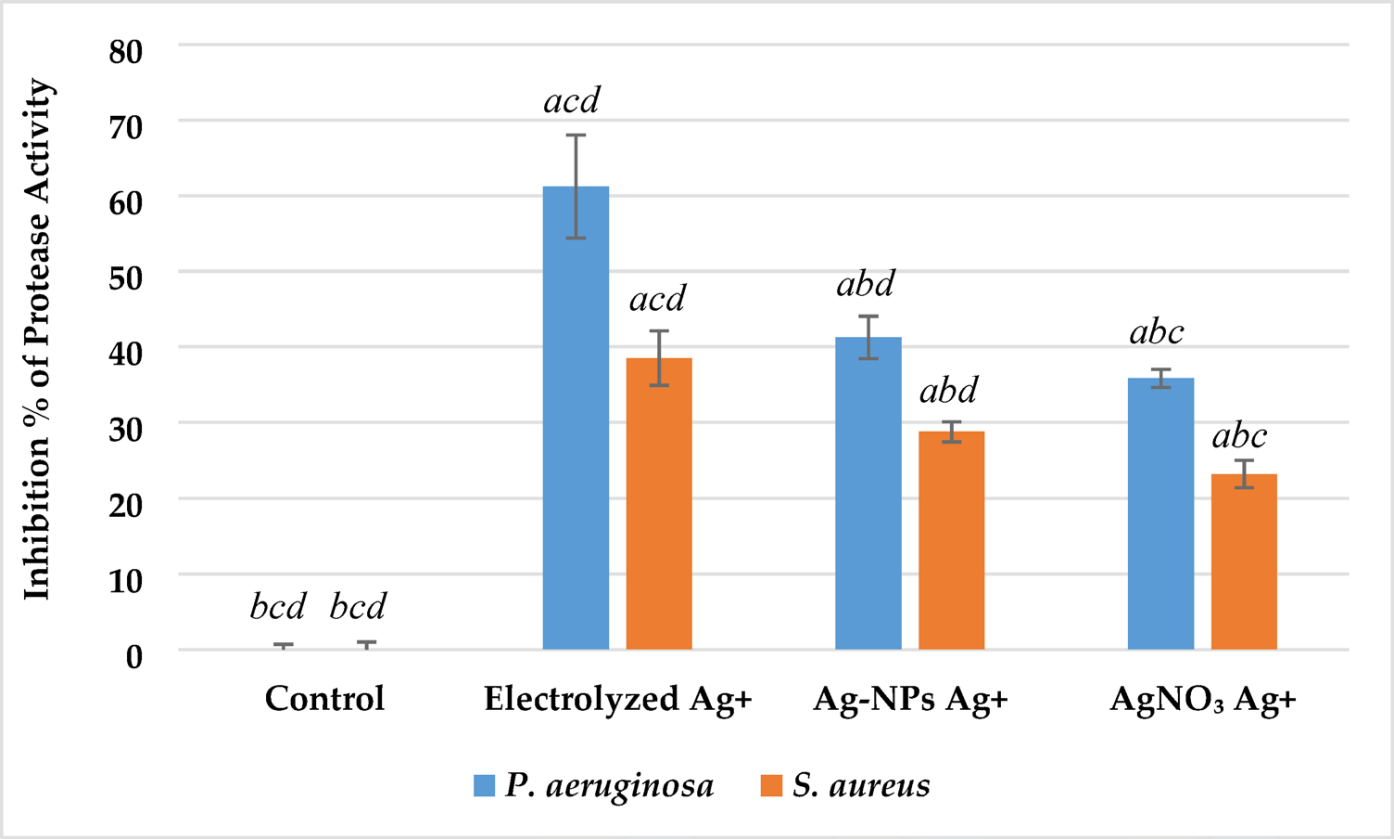
Effect of silver ion sources on the inhibition percentage of protease activity of *P. aeruginosa* and *S. aureus*. Data represent mean ± SD (n = 3). Statistical significance was determined by one-way ANOVA followed by Tukey’s HSD post-hoc test (p < 0.05). Different letters above the bars indicate significant differences among treatments: *a*—Control, *b*—Electrolyzed Ag⁺, *c*—Ag-NPs Ag⁺, and *d*—AgNO₃ Ag⁺.

*Impact on protease activity* Protease activity was markedly inhibited by all silver ion treatments compared to the control group, as presented in Fig. 13 (p < 0.05). In *P. aeruginosa*, electrolyzed Ag⁺ caused the highest inhibition of protease production (60.9 ± 5.7%), followed by Ag-NPs Ag⁺ (42.2 ± 2.5%) and AgNO₃ Ag⁺ (35.5 ± 2.2%). Similarly, in *S. aureus*, the highest inhibition was also observed with electrolyzed Ag⁺ (38.3 ± 3.2%), while Ag-NPs and AgNO₃ produced moderate reductions of 29.1 ± 1.8% and 23.4 ± 1.5%, respectively.

Electrolyzed Ag⁺ demonstrated significantly greater suppression of protease activity than both Ag-NPs and AgNO₃ in both bacterial strains (p < 0.05), suggesting a strong inhibitory effect on proteolytic virulence mechanisms.

**Fig. 14:**
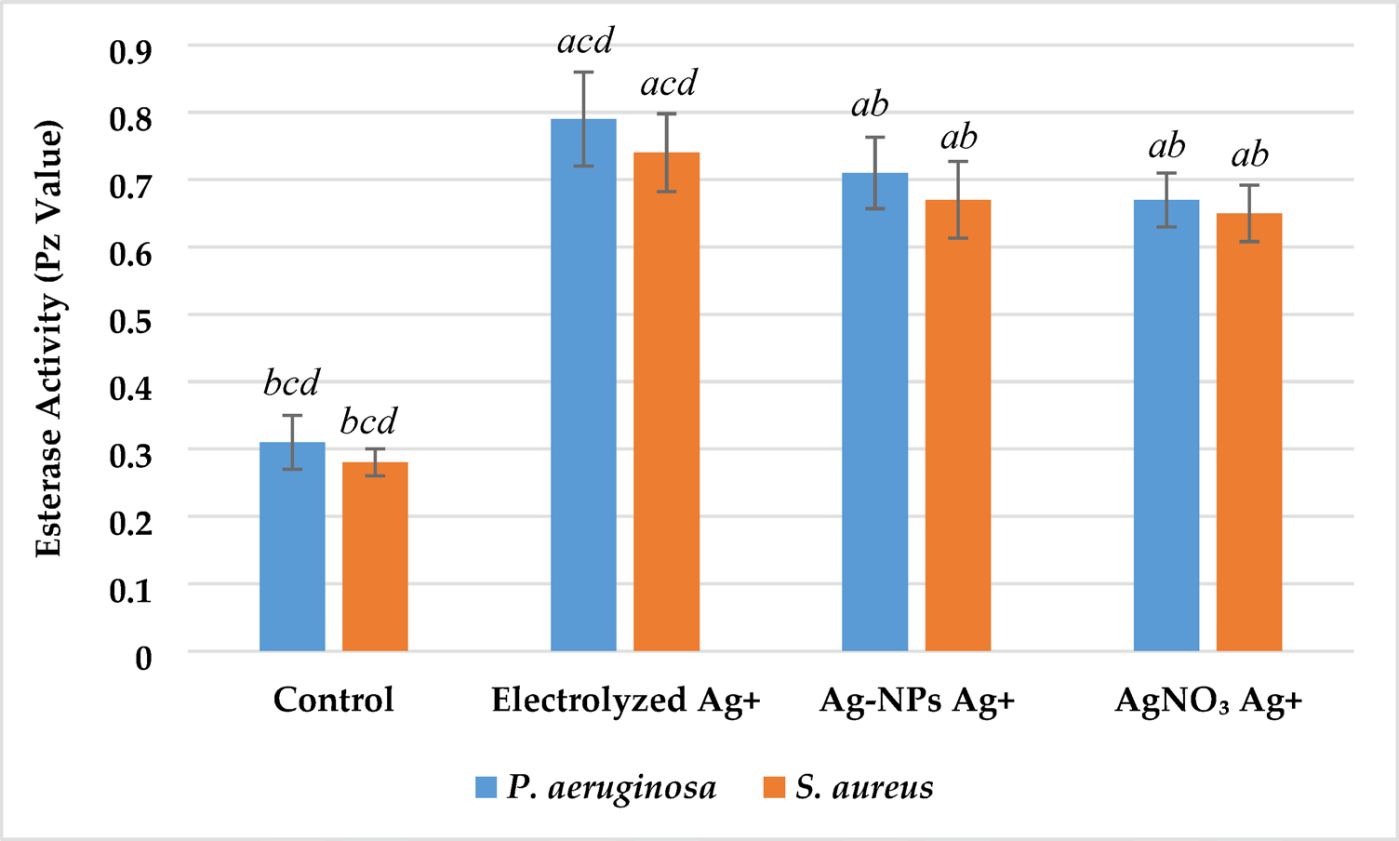
Effect of silver ion sources on the esterase activity Index (Pz) of *P. aeruginosa* and *S. aureus*. Data represent mean ± SD (n = 3). Statistical significance was determined by one-way ANOVA followed by Tukey’s HSD post-hoc test (p < 0.05). Different letters above the bars indicate significant differences among treatments: *a*—Control, *b*—Electrolyzed Ag⁺, *c*—Ag-NPs Ag⁺, and *d*—AgNO₃ Ag⁺.

*Impact on esterase activity* Among the tested silver ion sources, all treatments led to significant inhibition of esterase activity in both *P. aeruginosa* and *S. aureus*, as reflected by increased Pz values relative to the control (Fig. 14). Electrolyzed Ag⁺ exerted the most pronounced effect, with Pz values of 0.78 ± 0.06 for *P. aeruginosa* and 0.72 ± 0.05 for *S. aureus*, followed by Ag-NPs Ag⁺ (0.70 ± 0.05 and 0.66 ± 0.05, respectively) and AgNO₃ Ag⁺ (0.66 ± 0.04 and 0.64 ± 0.04, respectively). In contrast, the control groups showed strongly positive esterase activity with the lowest Pz values (0.31 ± 0.03 in *P. aeruginosa* and 0.27 ± 0.02 in *S. aureus*). The results indicated that all silver-treated groups demonstrated positive (moderate) esterase activity levels, confirming effective suppression, particularly with electrolyzed Ag⁺.

**Fig. 15:**
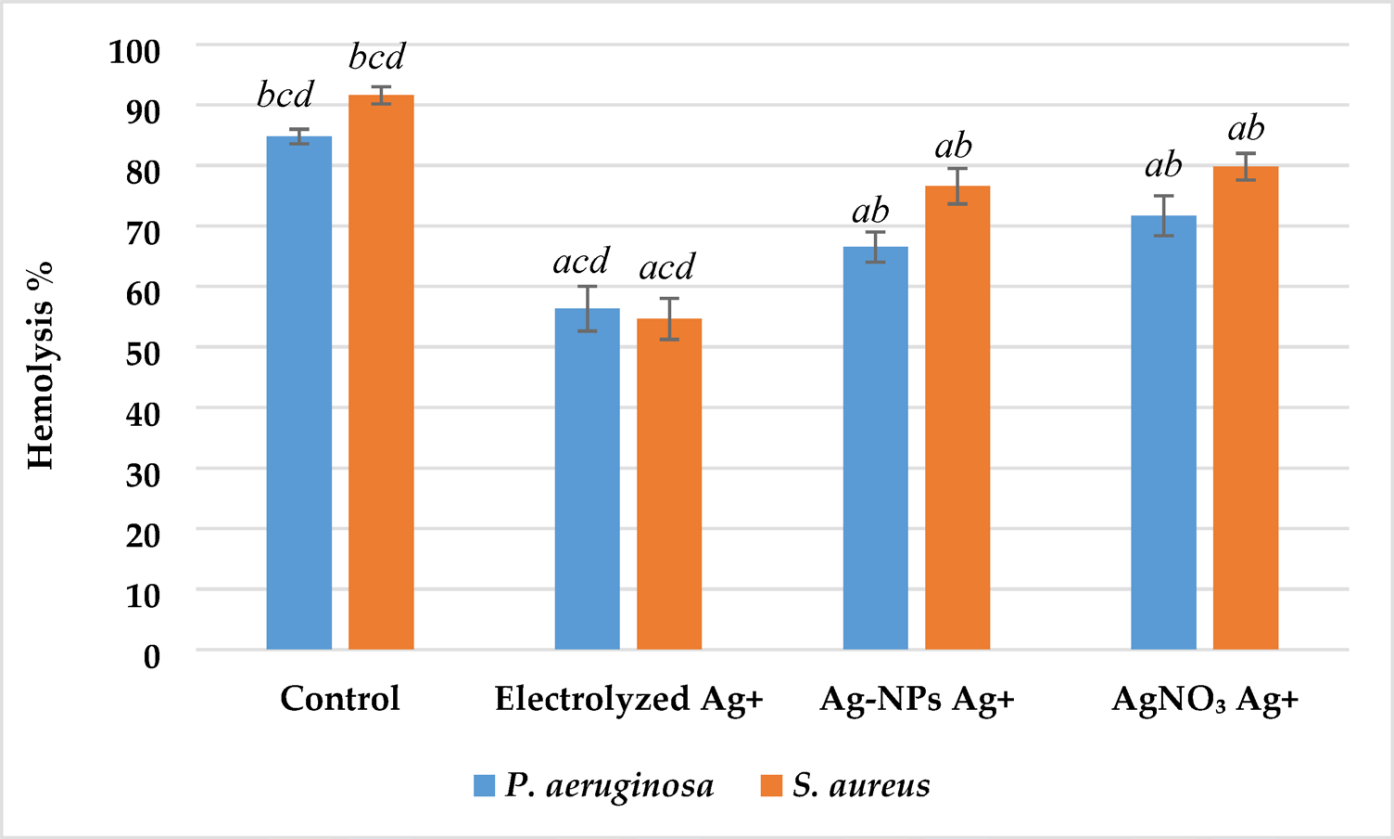
Effect of silver ion sources on the hemolysis percentage of *P. aeruginosa* and *S. aureus.* Data represent mean ± SD (n = 3). Statistical significance was determined by one-way ANOVA followed by Tukey’s HSD post-hoc test (p < 0.05). Different letters above the bars indicate significant differences among treatments: *a*—Control, *b*—Electrolyzed Ag⁺, *c*—Ag-NPs Ag⁺, and *d*—AgNO₃ Ag⁺.

*Impact on Hemolysin* Hemolytic activity was substantially reduced in all silver-treated groups compared to the untreated control (p < 0.01), indicating significant suppression of bacterial hemolysin production. The hemolysis percentage shown in Fig. 15 demonstrated that the strongest inhibition was observed with electrolyzed Ag⁺, resulting in hemolysis levels of approximately 56% in *P. aeruginosa* and 54% in *S. aureus*, which were highly significantly lower than the corresponding control values (85% and 92%, respectively; p < 0.001). Ag-NPs Ag⁺ and AgNO₃ Ag⁺ treatments also produced statistically significant reductions (p < 0.05–0.01), though less pronounced than electrolyzed Ag⁺. These findings demonstrate that all silver ion sources exert anti-virulence effects, with electrolyzed Ag⁺ showing the most significant inhibition of hemolysin-mediated cytotoxicity.

**Fig. 16:**
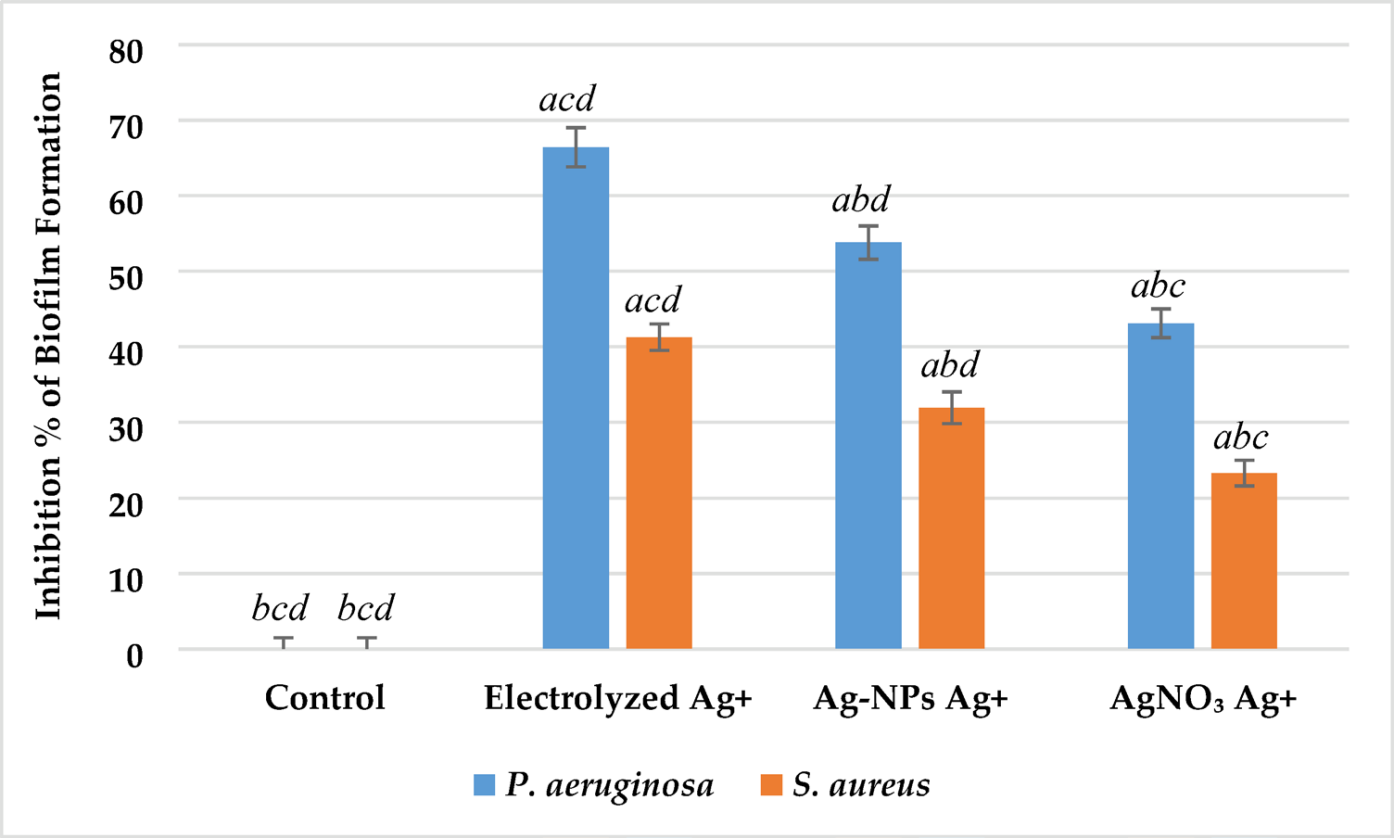
Effect of Silver Ion Sources on the Biofilm Formation Inhibition Percentage of *P. aeruginosa* and *S. aureus*. Data represent mean ± SD (n = 3). Statistical significance was determined by one-way ANOVA followed by Tukey’s HSD post-hoc test (p < 0.05). Different letters above the bars indicate significant differences among treatments: *a*—Control, *b*—Electrolyzed Ag⁺, *c*—Ag-NPs Ag⁺, and *d*—AgNO₃ Ag⁺.

*Impact on biofilm formation* Biofilm formation was significantly inhibited by all silver ion treatments compared to the untreated control (p < 0.01), as shown in Fig. 16. Electrolyzed Ag⁺ showed the most pronounced effect. In *P. aeruginosa*, biofilm inhibition reached approximately 66%, which was highly significantly greater than inhibition by Ag-NPs Ag⁺ (54%) and AgNO₃ Ag⁺ (44%) (p < 0.05). In *S. aureus*, similar trends were observed, with inhibition levels of 42%, 32%, and 24% for electrolyzed Ag⁺, Ag-NPs Ag⁺, and AgNO₃ Ag⁺, respectively. Untreated control groups showed minimal inhibition (< 2%) in both strains.

### SEM results

**Fig. 17:**
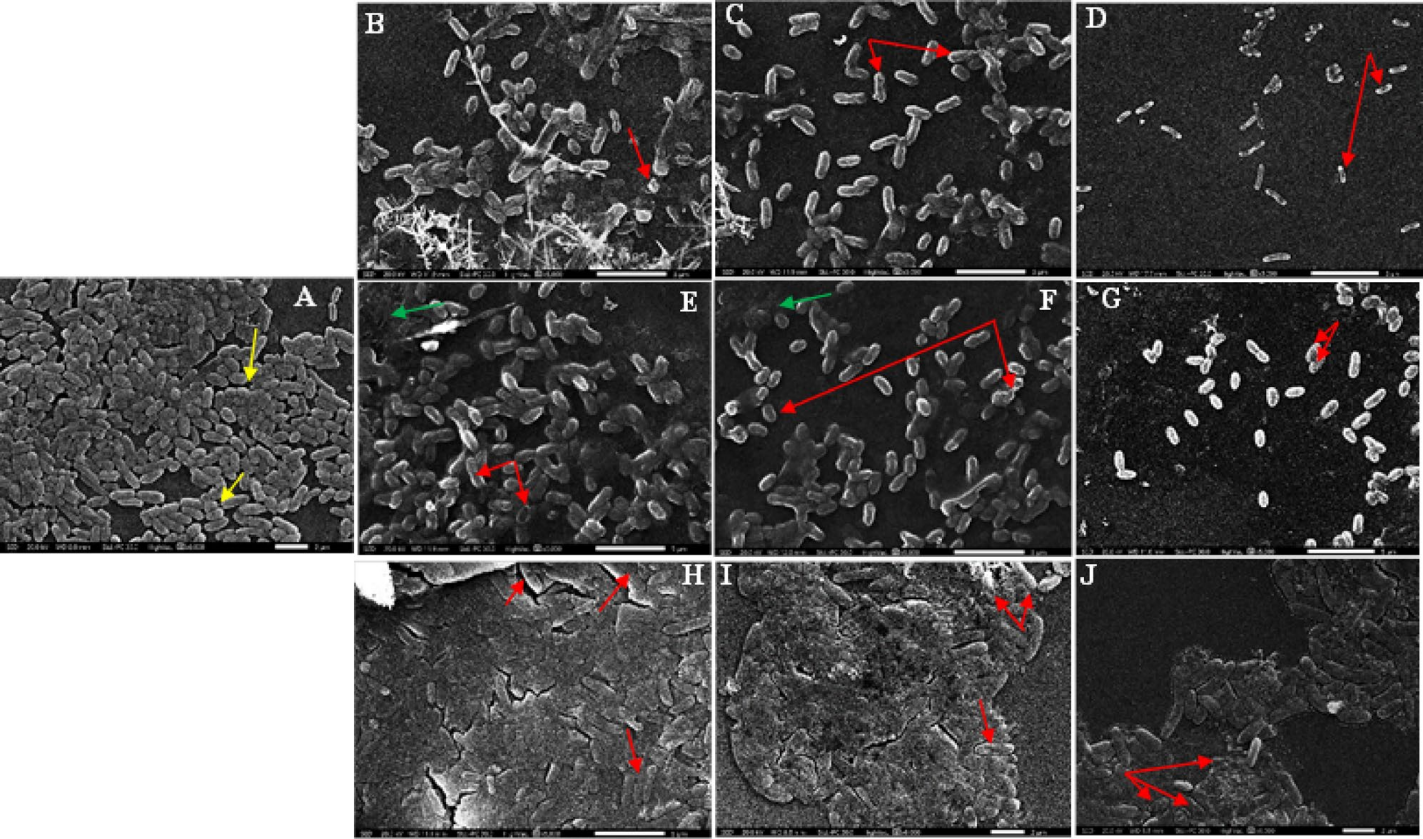
Morphological Changes Induced by Different Treatments Against *P. aeruginosa*. (**A**)- Untreated Control, (**B**, **C**, and **D**) –Treatments by Electrolyzed Ag⁺. (**E**, **F**, and **G**)- Treatments by Ag-NPs Ag⁺. (**H**, **I**, and **J**)-Treatment by AgNO3 Ag⁺. Biofilm Matrix with EPS Threads (Yellow Arrows), Cell Deformations (Red Arrows), and Disintegrated Biofilm (Green Arrows).

**Fig. 18:**
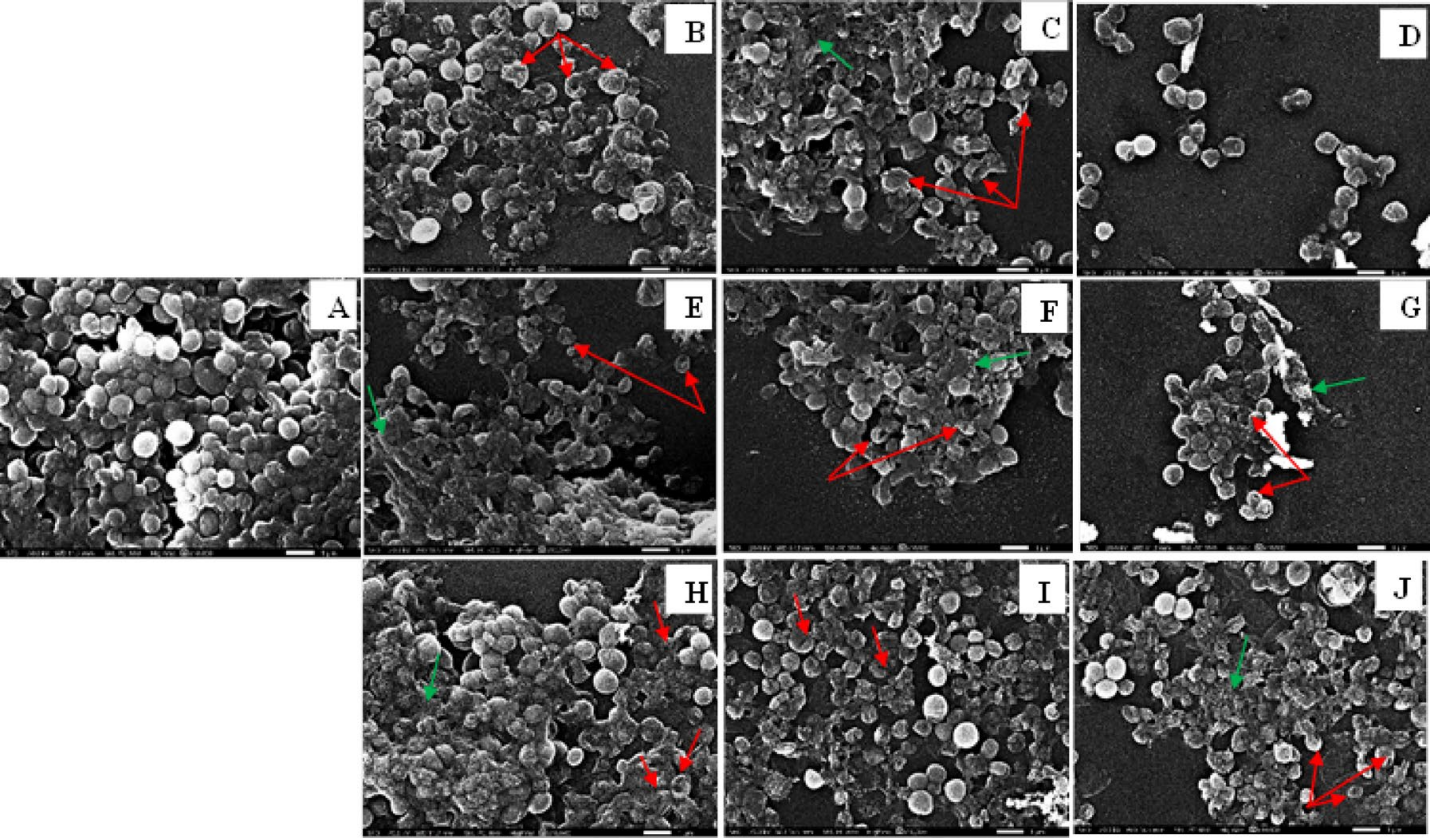
Morphological Changes Induced by Different Treatments Against *S. aureus*. (**A**)- Untreated Control, (**B**, **C**, and **D**) –Treatments by Electrolyzed Ag⁺. (**E**, **F**, and **G**)- Treatments by Ag-NPs Ag⁺. (**H**, **I**, and **J**)-Treatment by AgNO3 Ag⁺. Biofilm Matrix with EPS Threads (Yellow Arrows), Cell Deformations (Red Arrows), and Disintegrated Biofilm (Green Arrows).

Scanning electron microscopy (SEM) confirmed the morphological damage induced by silver ion treatments in both *P. aeruginosa* and *S. aureus*. In untreated control groups (Figs. 17A and 18A), both species maintained normal architecture: *P. aeruginosa* appeared as intact rods with visible septa, embedded in dense extracellular polymeric substance (EPS), while *S. aureus* retained its typical spherical shape, arranged in tight aggregates within a thick biofilm matrix. In contrast, treatment with electrolyzed Ag⁺ (Figs. 17B–D and 18B–D) caused extensive structural damage, including surface collapse, deep membrane crevices, and disrupted biofilm matrices. These structural alterations were most pronounced in *P. aeruginosa*, which also exhibited extensive membrane collapse and cell lysis.

Cells treated with Ag-NPs Ag⁺ (Figs. 17E–G and 18E–G) showed moderate surface distortions and partial EPS degradation in both strains, while AgNO₃ Ag⁺ (Figs. 17H–J and 18H–J) induced only mild deformation, with biofilm architecture largely preserved. These strain-consistent trends align with previous assays, further supporting the superior bactericidal effects of electrolyzed silver ions.

## Discussion

This study investigated the antibacterial and anti-virulence effects of silver ions derived from electrolyzed silver, silver nanoparticles (Ag-NPs), and silver nitrate against two clinically relevant pathogens, *Pseudomonas aeruginosa* and *Staphylococcus aureus*. To standardize efficacy comparisons across treatments, all experiments were conducted at a silver ion concentration of approximately 400 ppm. This concentration was chosen based on previous reports showing that MIC/MBC values for silver ions typically range between 1–100 ppm for most bacteria, but higher concentrations like 400 ppm are known to produce complete growth inhibition, particularly in complex matrices or biofilm states^28,29^.

Although all treatments were standardized to deliver 400 ppm of silver ions, the biological responses of *P. aeruginosa* and *S. aureus* varied markedly depending on the ion source. This clearly demonstrates that the antibacterial and anti-virulence activities of silver-based agents are not solely concentration-dependent but are also significantly influenced by the physicochemical characteristics conferred by the generation method. These findings support the concept that the ion origin, whether from electrolysis, nanoparticles, or salt, modulates bacterial responses and should be considered a critical design parameter in the development of advanced silver-based antimicrobials.

Among the tested treatments, electrolyzed Ag⁺ ions consistently demonstrated the most potent antibacterial effect, as shown by reductions in cultivability, metabolic activity, and disruption of membrane integrity (DNA, protein, and potassium leakage). These findings are consistent with prior reports indicating that electrochemically generated Ag⁺ ions display elevated reactivity and broader surface interactions^30,31^. In contrast, although Ag-NPs can also release silver ions, their antimicrobial effect depends on their size, shape, surface coating, and rate of ion release^32,33^. Our findings agree with Dziora et al. and Stabryla et al., who noted that Ag⁺ ions sometimes outperform Ag-NPs due to their direct interaction with cellular targets^7,31^.

Interestingly, silver nitrate, while chemically releasing the highest free Ag⁺, showed the lowest antimicrobial effect among the three sources. This could be attributed to precipitation, EPS binding, or reduced interaction kinetics, which limit Ag⁺ availability at the bacterial surface^34,35^.

The anti-virulence effects of electrolyzed silver were also significant. Notable inhibition was observed in pigment production (pyocyanin and staphyloxanthin), protease, esterase, hemolysin activity, and biofilm formation. These results align with studies by Hui et al. and Parai et al., who showed that silver treatments reduce virulence factors by disrupting quorum sensing and enzyme secretion^36,37^. Specifically, esterase activity shifted from strongly positive (Pz ≈ 0.3) to moderately positive levels (Pz ≈ 0.7) after silver treatment, indicating enzyme inhibition and decreased pathogenic potential^38^. Biofilm formation was similarly affected, with electrolyzed Ag⁺ showing the greatest reduction (~ 66% in *P. aeruginosa*), followed by Ag-NPs and silver nitrate. SEM images confirmed extensive biofilm disruption and cell collapse, especially in *P. aeruginosa*. These morphological changes further support prior findings that silver ions can penetrate biofilms and damage EPS structures^35,39^.

A consistent observation throughout the study was that *P. aeruginosa* showed greater susceptibility than *S. aureus*. This is due to *P. aeruginosa*'s thinner peptidoglycan layer and higher outer membrane permeability, which make it more vulnerable to Ag⁺-induced membrane destabilization^29,40^. Additionally, differences in stress response systems and metal ion transporters may influence the species-specific effectiveness of silver treatments^31,37^.

While some previous studies have concluded that Ag-NPs outperform Ag⁺ due to sustained release and nanoparticle-cell interactions^32^, our findings suggest that the way Ag⁺ is generated plays a more crucial role in determining antimicrobial effectiveness. However, our results show that the method of Ag⁺ production and delivery (such as through electrolysis) significantly impacts its bioactivity, even at identical concentrations. The superior performance of electrolyzed Ag⁺ over Ag-NPs and AgNO₃ is likely due to improved bioavailability, oxidative interactions, and reactivity with bacterial targets. Prior research has demonstrated that electrically generated silver ions cause significant membrane disruption and induce VBNC states in both S. aureus and E. coli^41^. Additionally, across various bacterial species, silver ions induce ROS-mediated oxidative stress, with superoxide radicals accounting for nearly half of the bactericidal effect^42^. This mechanistic insight supports our observations that electrolyzed Ag⁺ results in greater permeability, leakage, and suppression of virulence compared to Ag-NPs or AgNO₃.

The strong performance of electrolyzed Ag⁺ at standardized concentrations justifies its selection for developing next-generation antimicrobial coatings. It also indicates possible uses in clinical wound dressings, surface disinfectants, and water treatment systems, especially in environments challenged by bacterial biofilms or resistant strains. Compared to silver nanoparticles, electrolyzed silver ions may pose fewer long-term ecological risks, as they can degrade or precipitate more easily, potentially lowering silver buildup in the environment^43^. Future research should further explore the molecular interactions between electrolyzed Ag⁺ and specific bacterial proteins or membranes using proteomic or transcriptomic profiling.

While this study provides a direct comparative evaluation of electrolyzed Ag⁺, Ag-NPs, and AgNO₃ at an equivalent concentration, it is limited by the *in-vitro* design and the use of a single standardized Ag⁺ level (400 ppm). Although basic physicochemical parameters such as pH and conductivity were measured during electrolysis and found to change only slightly, other variables such as reactive oxygen species (ROS) formation and long-term ion stability were not systematically quantified. Cytotoxicity toward mammalian cells and in vivo validation were also beyond the scope of this work and should be explored in future studies. Although electrolyzed Ag⁺ demonstrated superior antibacterial and anti-virulence effects under standardized in vitro conditions, further in vivo studies are required before clinical translation.

## Conclusions

The present study showed that the antibacterial and anti-virulence effects of silver ions are greatly affected by how they are generated. Among the tested sources, electrolyzed Ag⁺ consistently performed better than silver nanoparticles and silver nitrate in stopping the growth, virulence, and biofilm formation of *Pseudomonas aeruginosa* and *Staphylococcus aureus* at a standard concentration of 400 ppm.

Electrolyzed Ag⁺ caused significant disruption to bacterial viability, membrane integrity, and metabolic activity, with strong inhibition of motility, enzyme activity, and extracellular matrix production. These effects were particularly notable in *P. aeruginosa*, emphasizing species-specific differences in susceptibility to oxidative stress and ion penetration. The findings highlight that the method of ion delivery, beyond just concentration, plays a crucial role in antimicrobial effectiveness.

These findings suggest that electrolyzed silver ions could be further explored for the development of advanced antimicrobial biomaterials, particularly for applications such as medical device coatings, wound dressings, and surgical implants. Their powerful biofilm-disrupting and anti-virulence properties suggest potential for reducing chronic infections and reliance on antibiotics. Future research should focus on evaluating in vivo performance, long-term safety, and integration into multifunctional or composite biomaterial systems.

## Data Availability

All data generated or analyzed during this study are included in this published article. The raw data supporting the conclusions of this article will be made available by the corresponding authors on request.
